# NEK2 plays an essential role in porcine embryonic development by maintaining mitotic division and DNA damage response via the Wnt/β‐catenin signalling pathway

**DOI:** 10.1111/cpr.13626

**Published:** 2024-03-01

**Authors:** Se‐Been Jeon, Pil‐Soo Jeong, Hyo‐Gu Kang, Min Ju Kim, Ji Hyeon Yun, Kyung Seob Lim, Bong‐Seok Song, Sun‐Uk Kim, Seong‐Keun Cho, Bo‐Woong Sim

**Affiliations:** ^1^ Futuristic Animal Resource & Research Center (FARRC) Korea Research Institute of Bioscience and Biotechnology (KRIBB) Cheongju Republic of Korea; ^2^ Department of Animal Science, College of Natural Resources & Life Science Pusan National University Miryang Republic of Korea; ^3^ Department of Animal Science and Biotechnology, College of Agriculture and Life Science Chungnam National University Daejeon Republic of Korea; ^4^ Department of Animal BioScience, School of Animal Life Convergence Hankyong National University Ansung Republic of Korea; ^5^ Department of Functional Genomics University of Science and Technology Daejeon Republic of Korea; ^6^ Department of Animal Science, Life and Industry Convergence Research Institute (RICRI), College of Natural Resources & Life Science Pusan National University Miryang Republic of Korea

## Abstract

NIMA‐related kinase 2 (NEK2) is a serine/threonine protein kinase that regulates mitosis and plays pivotal roles in cell cycle regulation and DNA damage repair. However, its function in porcine embryonic development is unknown. In this study, we used an NEK2‐specific inhibitor, JH295 (JH), to investigate the role of NEK2 in embryonic development and the underlying regulatory mechanisms. Inhibition of NEK2 after parthenogenesis activation or in vitro fertilization significantly reduced the rates of cleavage and blastocyst formation, the numbers of trophectoderm and total cells and the cellular survival rate compared with the control condition. NEK2 inhibition delayed cell cycle progression at all stages from interphase to cytokinesis during the first mitotic division; it caused abnormal nuclear morphology in two‐ and four‐cell stage embryos. Additionally, NEK2 inhibition significantly increased DNA damage and apoptosis, and it altered the expression levels of DNA damage repair‐ and apoptosis‐related genes. Intriguingly, NEK2 inhibition downregulated the expression of β‐catenin and its downstream target genes. To validate the relationship between Wnt/β‐catenin signalling and NEK2 during porcine embryonic development, we cultured porcine embryos in JH‐treated medium with or without CHIR99021, a Wnt activator. CHIR99021 co‐treatment strongly restored the developmental parameters reduced by NEK2 inhibition to control levels. Our findings suggest that NEK2 plays an essential role in porcine embryonic development by regulating DNA damage repair and normal mitotic division via the Wnt/β‐catenin signalling pathway.

## Graphical abstract

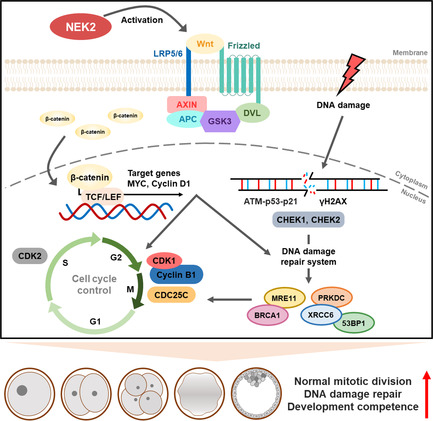

## 1 INTRODUCTION

In mammals, successful completion of mitotic cell division that continues to cleavage and then blastocyst development is essential for preimplantation embryonic development. However, embryonic development can be arrested at any time from fertilization to the blastocyst stage. Arrested embryos undergo degeneration, including fragmentation or lysis, which is a major cause of female infertility.^1^ Chromosomal abnormalities, gene variants, DNA damage and dysregulation of the cell cycle and signalling pathway have been identified as the main causes of embryonic arrest.^2^ Therefore, an understanding of the underlying regulatory mechanisms and signalling pathways that govern preimplantation embryonic development is important for basic reproductive biology and clinical applications.

In eukaryotic cells, the cell cycle is a series of events in which genetic information is replicated and passed to the next generation: two separate genetically identical daughter cells. To preserve genome integrity, cells have developed complex checkpoint systems that ensure DNA replication and proper chromosome segregation.^3^ In response to DNA damage, cells commonly activate checkpoint systems that induce cell cycle delay or arrest to repair their DNA.^4^ Disruption of the checkpoint systems may limit or prevent DNA repair, leading to mitotic defects such as chromosomal abnormalities, apoptosis and/or programmed cell death.^5^, ^6^ In particular, the G2/M checkpoint helps to maintain genetic stability by blocking damaged or incompletely replicated DNA from entering mitosis until repair is complete.^7^ Cell cycle regulators such as checkpoint kinases, p53, polo‐like kinases, Aurora kinases and never in mitosis gene A (NIMA)‐related kinases are involved in controlling the G2/M checkpoint and regulating centrosome segregation.^8^

The NIMA‐related kinases (i.e., NEKs) constitute a group of serine/threonine kinases initially described in *Aspergillus nidulans*; they serve as essential regulators of mitosis.^9^ NEKs are involved in various cellular events during cell cycle progression, checkpoint regulation and differentiation; they have recently been associated with the DNA damage response.^10^, ^11^ In mammals, there are 11 NEKs (NEK1 through NEK11); NEK2 is the best‐characterized member of these proteins.^12^ There is increasing evidence that NEK2 participates in multiple cellular processes through the phosphorylation of specific groups of substrates and regulation of signalling pathways.^13^, ^14^ NEK2 serves as a regulator of mitosis, including mitotic initiation, chromatin condensation and spindle organization.^13^ NEK2 also contributes to centrosome segregation during the G2/M transition.^15^ In addition to its job as a centrosome kinase, NEK2 is a multifunctional protein that plays pivotal roles in development, DNA damage repair and cell cycle regulation.^16^ Although previous studies have investigated the function and cellular localization of NEK2 in vertebrate reproductive systems,^17^, ^18^, ^19^ the involvement of NEK2 and the associated mechanisms during early embryogenesis have not been clarified.

The Wnt signalling pathway regulates several biological signals, including cell proliferation and differentiation, and plays an essential role in all stages of vertebrate embryonic development.^20^, ^21^, ^22^ The canonical Wnt signalling pathway is a β‐catenin‐dependent pathway that becomes activated upon binding to the Wnt‐Frizzled‐LRP5/6 complex. β‐catenin is stabilized with the recruitment of Dishevelled and Axin scaffolding proteins. The accumulated β‐catenin then moves to the nucleus and binds to T‐cell/lymphoid enhancer transcription factors (TCF/LEF) to activate the expression of Wnt target genes such as cyclin D1 and c‐Myc.^23^ However, Wnt inactivation results in continued β‐catenin degradation by the β‐catenin destruction complex consisting of GSK3, APC and AXIN.^23^ Although the importance of the Wnt/β‐catenin signalling pathway in multiple cell types is well‐known,^24^, ^25^ there has been minimal research concerning this signalling pathway in porcine early embryogenesis.

A previous study has shown that β‐catenin is a physiological substrate of the NEK2 and is crucial for regulating centrosome separation during mitosis.^26^ NEK2 prevents β‐catenin ubiquitination and degradation by phosphorylating the same regulatory site in the N‐terminus of β‐catenin as GSK3β.^27^ Several studies have reported that overexpression or knockdown of NEK2 altered β‐catenin levels and expression of Wnt downstream target genes c‐Myc and cyclin D1,^28^, ^29^, ^30^ indicating that NEK2 regulates the Wnt/β‐catenin signalling pathway. However, the mechanism and evaluation between NEK2 and Wnt/β‐catenin signalling pathway have not been elucidated in porcine early embryonic development. In the present study, we used the specific inhibitor JH295 (an irreversible cysteine‐targeted inhibitor that inhibits cellular NEK2 through alkylation of Cys22)^31^ to investigate the role of NEK2 during porcine early embryogenesis and the potential underlying regulatory mechanisms by observing the subcellular localization and expression patterns of NEK2 in porcine embryos and investigating the effects of NEK2 inhibition on embryonic development and mitotic division. Specifically, we assessed embryo developmental kinetics and blastocyst quality while monitoring cell cycle progression, DNA damage response and apoptosis in an attempt to determine the relationship between NEK2 and the Wnt/β‐catenin signalling pathway during early embryonic development in porcine.

## 2 MATERIALS AND METHODS

See Supporting Information for more details.

## 3 RESULTS

### 3.1 Dynamic expression pattern and subcellular localization of NEK2 during porcine embryonic development

**FIGURE 1:**
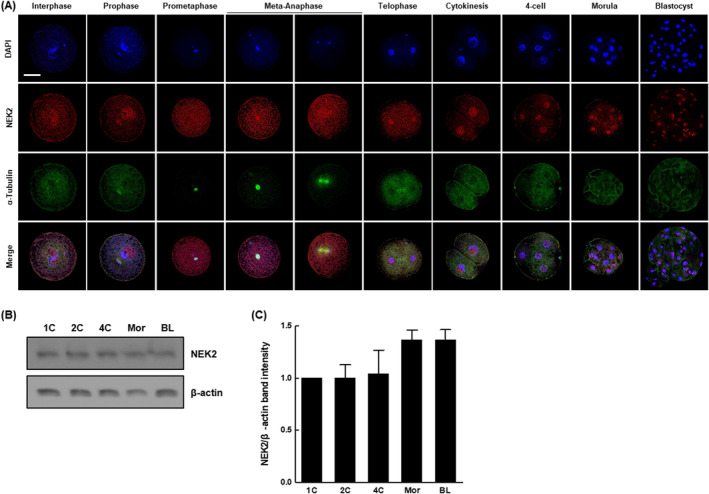
Subcellular localization and expression pattern of NIMA‐related kinase 2 (NEK2) in porcine embryogenesis. (A) Subcellular localization of NEK2 in porcine embryos at various stages. Bar = 100 μm. (B) Representative images of NEK2 Western blotting results and (C) quantification of NEK2 band intensity (*n* = 3 per group). Data are derived from at least three independent experiments; different superscripts indicate significant differences (*p* < 0.05). 1C, one‐cell stage; 2C, two‐cell stage; 4C, four‐cell stage; BL, blastocyst stage; Mor, morula stage.

To determine the role of NEK2 in porcine early embryogenesis, we first examined its expression pattern and subcellular localization at various stages of development. NEK2 was detected in the nucleus and cytoplasm during the first mitotic division, including interphase, prophase, pro‐metaphase, meta‐anaphase and telophase (Figure 1A). Additionally, NEK2 was consistently localized to the nucleus and cytoplasm from cytokinesis to the blastocyst stage. We then assessed the protein levels of NEK2 at various stages of porcine embryo development. Protein levels of NEK2 were continuously expressed at all stages of embryonic development (Figure 1B,C).

### 3.2 NEK2 inhibition decreases the developmental competence of porcine PA embryos

**FIGURE 2:**
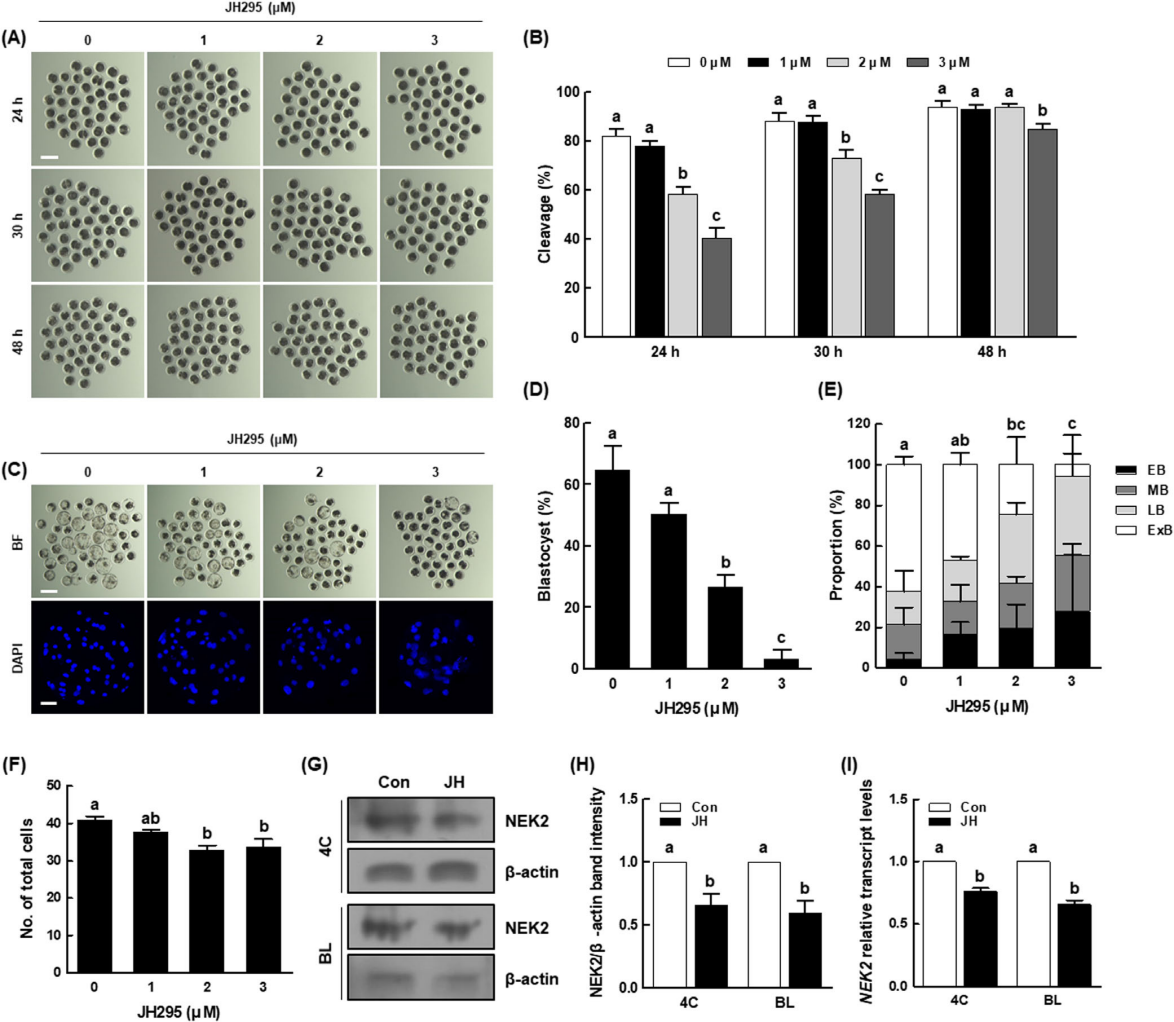
Effects of NEK2 inhibition during porcine parthenogenetic activation (PA) embryo development. (A) Representative images of cleavage and (B) cleavage rates of porcine PA embryos at 24, 30 and 48 h in the indicated groups (0; *n* = 200, 1; *n* = 200, 2; *n* = 200, 3; *n* = 199). Bar = 200 μm. (C) Representative bright‐field images of embryos (upper, Bar = 200 μm) and nuclear‐stained images (lower, Bar = 50 μm) of blastocysts in the indicated groups. (D) Blastocyst formation rates in the indicated groups (0; *n* = 200, 1; *n* = 200, 2; *n* = 200, 3; *n* = 199). (E) Proportions of various blastocyst stages in the indicated groups (0; *n* = 66, 1; *n* = 58, 2; *n* = 25, 3; *n* = 14). (F) Total cell numbers in the indicated groups (*n* = 24 per group). (G) Representative images of NEK2 Western blotting results and (H) quantification of NEK2 band intensity in the indicated groups (*n* = 3 per group). (I) qRT‐PCR results for transcript levels of *NEK2* genes in the indicated groups (*n* = 3 per group). Data are derived from at least three independent experiments; different superscripts indicate significant differences (*P* < 0.05). 4C, four‐cell stage; BL, blastocyst stage.

To investigate the effects of NEK2 inhibition on porcine early embryogenesis, we cultured PA embryos in IVC medium treated with various concentrations of JH (0, 1, 2 and 3 μM), then evaluated the rates of cleavage and blastocyst formation. After 24 and 30 h of culture, the cleavage rate had significantly decreased in a dose‐dependent manner (Figure 2A,B and Table S2). After 48 h of culture, the number of embryos in the 2 μM JH group reached the number in the control (Figure 2A,B and Table S2); however, the proportion of three‐ to four‐cell stage embryos was significantly lower than in the control (Figure S1). Intriguingly, the proportion of lysed embryos was initially significantly higher in the 2 μM JH group than in the control at day 3 and remained consistently high until day 6 (Figure S1); additionally, the blastocyst formation rate, the proportion of expanded blastocysts and the total cell number were considerably lower in the 2 μM JH group than in the control (Figure 2C–F and Tables S2 and S3). Moreover, developmental kinetics during the preimplantation period were delayed in the 2 μM JH group compared with the control (Figure S1). The 2 μM JH treatment significantly decreased the protein and mRNA expression levels of NEK2 in four‐cell stage embryos and blastocysts compared with the control, validating the effectiveness of NEK2 inhibition (Figure 2G–I). Based on these data, 2 μM of JH was used as the concentration for subsequent experiments.

**FIGURE 3:**
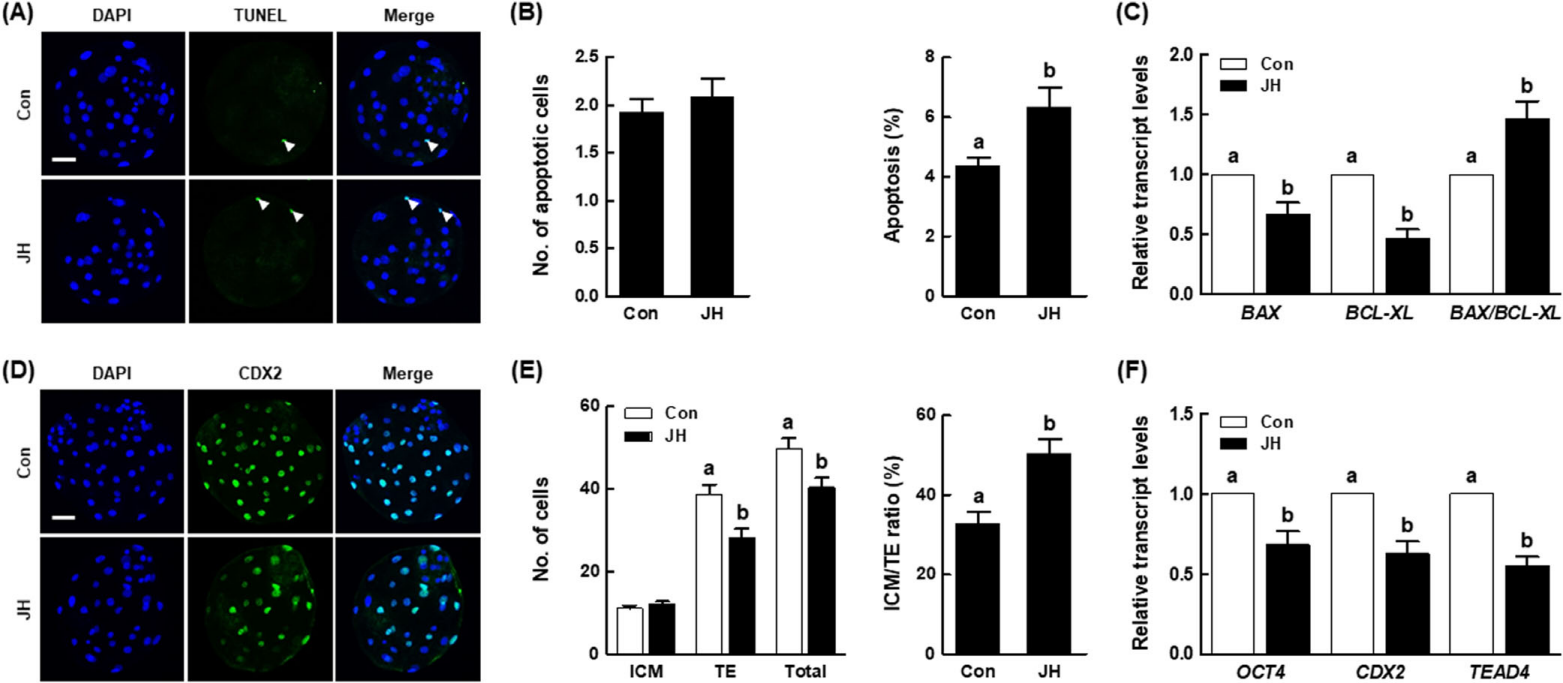
Effects of NEK2 inhibition on porcine PA blastocyst quality. (A) Representative images of TdT‐mediated dUTP nick‐end labelling (TUNEL) of blastocysts (Bar = 50 μm). Embryos were subjected to TUNEL staining (green, white arrow) and nuclear staining (blue). (B) Quantification of the number (left) and proportion (right) of apoptotic cells in the indicated groups (*n* = 50 per group). (C) qRT‐PCR results for transcript levels of apoptosis‐related genes and *BAX/BCL‐XL* ratio in blastocysts (*n* = 5 per group). (D) Representative images of CDX2 staining of blastocysts (Bar = 50 μm). (E) Numbers of inner cell mass (ICM), trophectoderm (TE), and total cells (left), as well as ICM/TE ratios (right), in the indicated groups (*n* = 40 per group). (F) qRT‐PCR results for transcript levels of developmental potential‐related genes in blastocysts (*n* = 5 per group). Data are derived from at least three independent experiments; different superscripts indicate significant differences (*P* < 0.05).

In our investigation of blastocyst quality using the TUNEL assay and CDX2 staining, the JH group showed no difference in the number of apoptotic cells; however, the apoptosis rate was significantly higher than in the control (Figure 3A,B and Table S4). The expression levels of pro‐apoptosis‐related (*BAX*) and anti‐apoptosis‐related (*BCL‐XL*) genes were significantly reduced in the JH group, and the *BAX*/*BCL‐XL* ratio was significantly increased compared with the control (Figure 3C). There was no difference in the number of inner cell mass (ICM) cells between the control and JH groups; however, the numbers of trophectoderm (TE) cells and total cells were significantly lower in the JH group, leading to an abnormal ICM to TE ratio (Figure 3D,E and Table S5). Compared with the control, the JH group showed a significant reduction in the expression levels of genes involved in developmental potential (*OCT4*, *CDX2* and *TEAD4*), as shown in (Figure 3F).

### 3.3 NEK2 inhibition decreases the developmental competence of porcine IVF embryos

**FIGURE 4:**
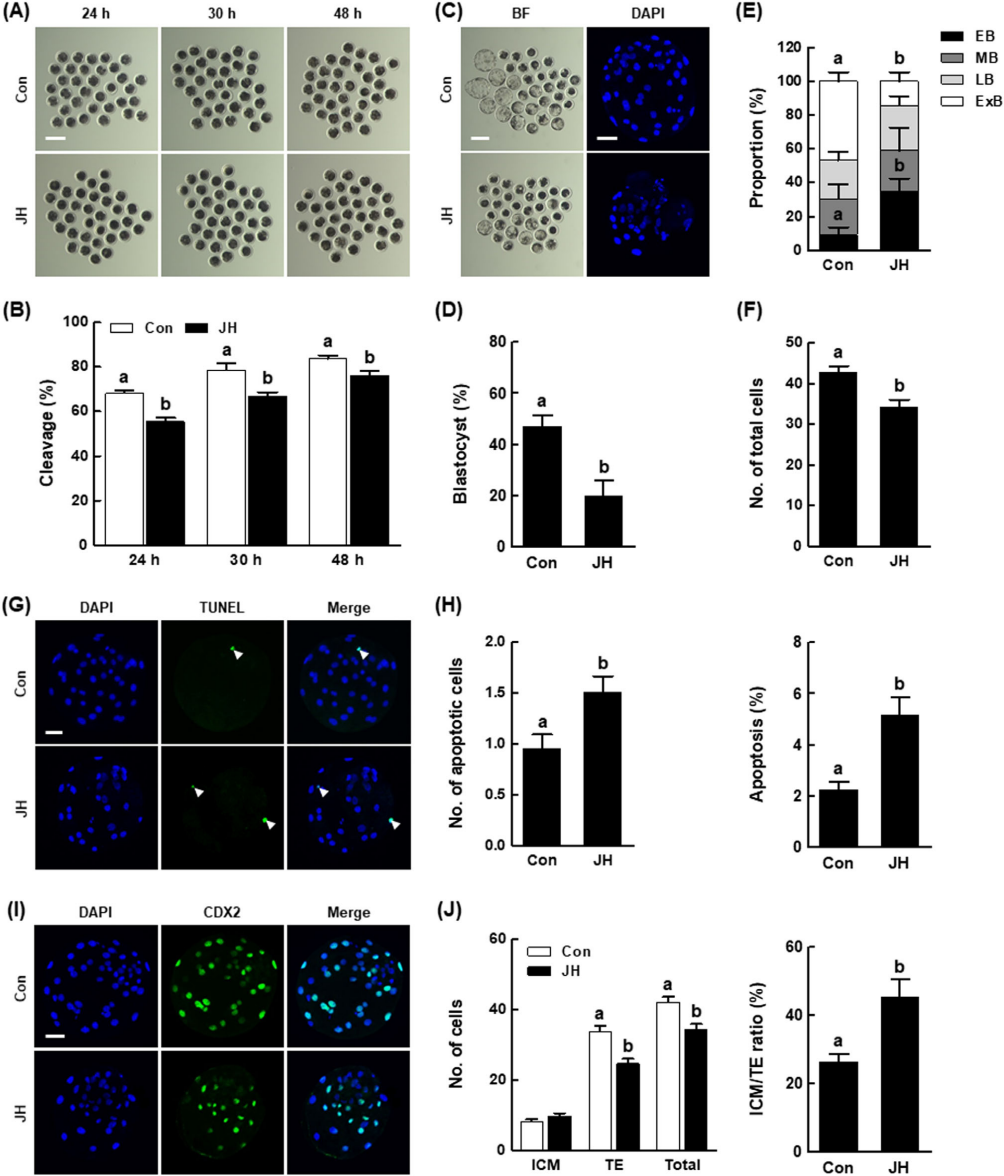
Effects of NEK2 inhibition on blastocyst development and quality among porcine in vitro fertilization (IVF) embryos. (A) Representative images of cleavage and (B) cleavage rates of porcine IVF embryos at 24, 30 and 48 h in the indicated groups (Con; *n* = 141, JH; *n* = 142). (C) Representative bright‐field images of embryos (left, Bar = 200 μm) and nuclear‐stained images (right, Bar = 50 μm) of blastocysts in the indicated groups. (D) Blastocyst formation rates in the indicated groups (Con; *n* = 141, JH; *n* = 142). (E) Proportions of various blastocyst stages in the indicated groups (Con; *n* = 49, JH; *n* = 25). (F) Total cell numbers in the indicated groups (*n* = 40 per group). (G) Representative images of TUNEL of blastocysts (Bar = 50 μm). Embryos were subjected to TUNEL staining (green, white arrow) and nuclear staining (blue). (H) Quantification of the number (left) and proportion (right) of apoptotic cells in the indicated groups (*n* = 47 per group). (I) Representative images of CDX2 staining of blastocysts (Bar = 50 μm). (J) Numbers of ICM, TE and total cells (left), as well as ICM/TE ratios (right), in the indicated groups (*n* = 33 per group). Data are derived from at least three independent experiments; different superscripts indicate significant differences (*P* < 0.05).

We performed further investigations concerning the effect of NEK2 inhibition on the developmental competence of IVF embryos. Consistent with PA results, the JH group showed a significant reduction in cleavage rates after 24, 30 and 48 h of culture, along with delayed developmental kinetics during the preimplantation period, compared with the control (Figure 4A,B and Figure S2 and Table S6). Furthermore, the proportion of lysed embryos in the JH group was consistently high from day 2 to day 6 (Figure S2). The blastocyst formation rate, proportion of expanded blastocysts and total cell number were considerably decreased in the JH group compared with the control (Figure 4C–F and Tables S6 and S7). Next, we evaluated the quality of blastocysts derived from JH‐treated IVF embryos. The JH group showed a significantly higher number of apoptotic cells and increased rate of apoptosis, compared with the control (Figure 4G,H and Table S8). Moreover, the JH group showed reduced numbers of TE and total cells, and an abnormal ICM to TE ratio, compared with the control (Figure 4I,J and Table S9). These results suggest that NEK2 plays an important role in porcine early embryogenesis.

### 3.4 NEK2 inhibition disrupts cell cycle progression during the first mitotic division of porcine embryos

**FIGURE 5:**
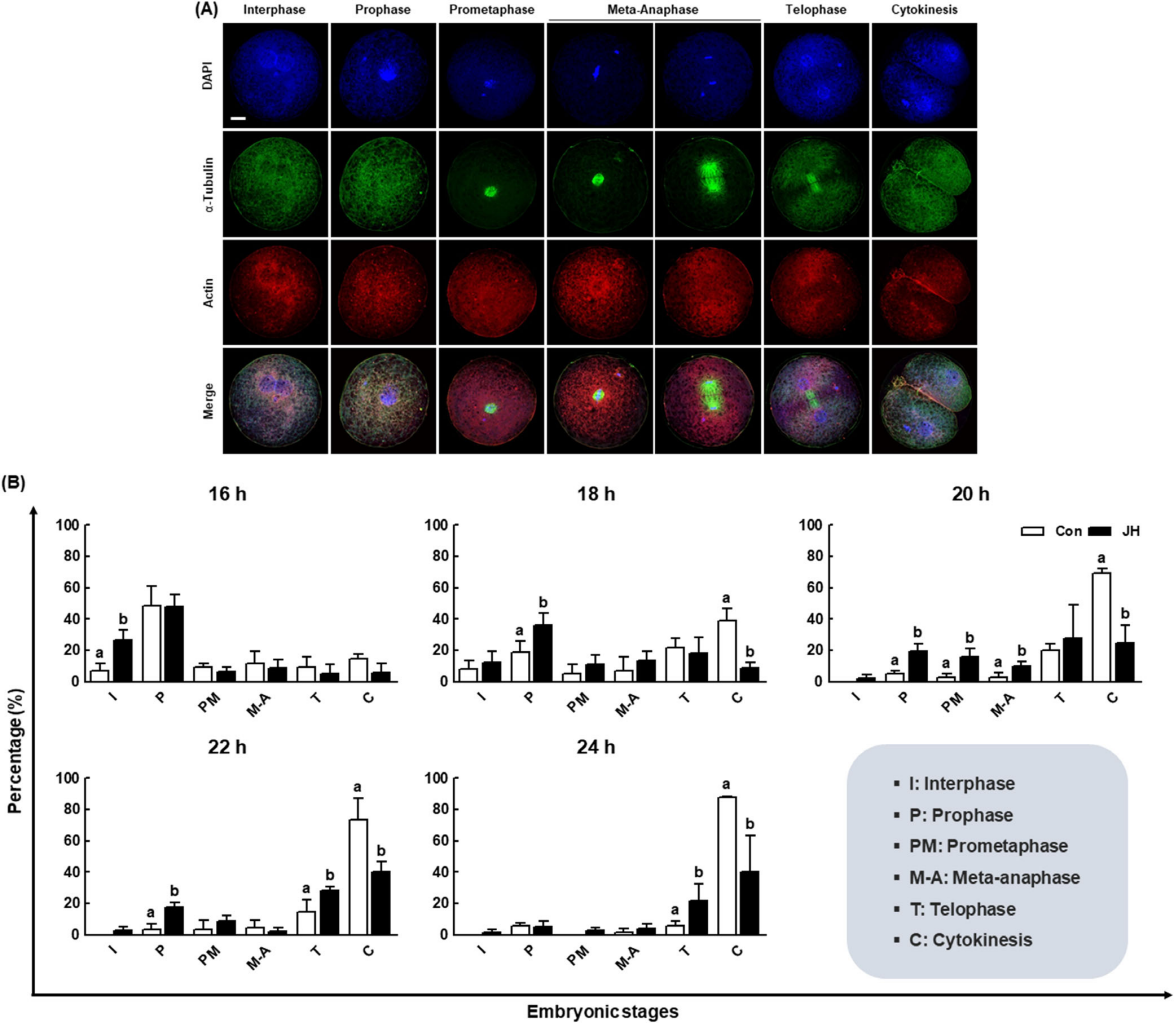
Effects of NEK2 inhibition on cell cycle progression during first mitotic division of porcine PA embryos. (A) Dynamic distribution of the cytoskeleton and nuclei at various stages of the first mitotic division in porcine embryos. Bar = 50 μm. (B) Quantification of cell cycle progression analysis during the first mitotic division in porcine embryos in the indicated groups (Con; *n* = 416, JH; *n* = 437). Data are derived from at least three independent experiments; different superscripts indicate significant differences (*P* < 0.05).

To determine when NEK2 inhibition delays porcine early embryonic development, we observed the mitotic division of embryos via time‐lapse monitoring over 48 h of culture. We found that JH‐treated embryos exhibited delays during their first and second mitotic divisions (Figure S3), suggesting that NEK2 inhibition interrupts normal cell cycle progression. Based on these results, we investigated the dynamic distribution of the cytoskeleton at various stages of the first mitotic division, using immunofluorescence staining to further analyse the effects of NEK2 inhibition on cell cycle progression in porcine embryos. According to the chromosomal and microtubule morphologies, we classified the embryonic stages into interphase, prophase, pro‐metaphase, meta‐anaphase, telophase and cytokinesis (Figure 5). At 16 and 18 h, the JH group had larger proportions of interphase‐ and prophase‐stage embryos than the control, respectively. Moreover, the proportion of cytokinesis‐stage embryos was consistently lower in the JH group than in the control from 18 to 24 h. Therefore, these results demonstrate that NEK2 inhibition disturbs normal cell cycle progression, including the first mitotic division.

### 3.5 NEK2 inhibition causes abnormal nucleation status in porcine embryos

**FIGURE 6:**
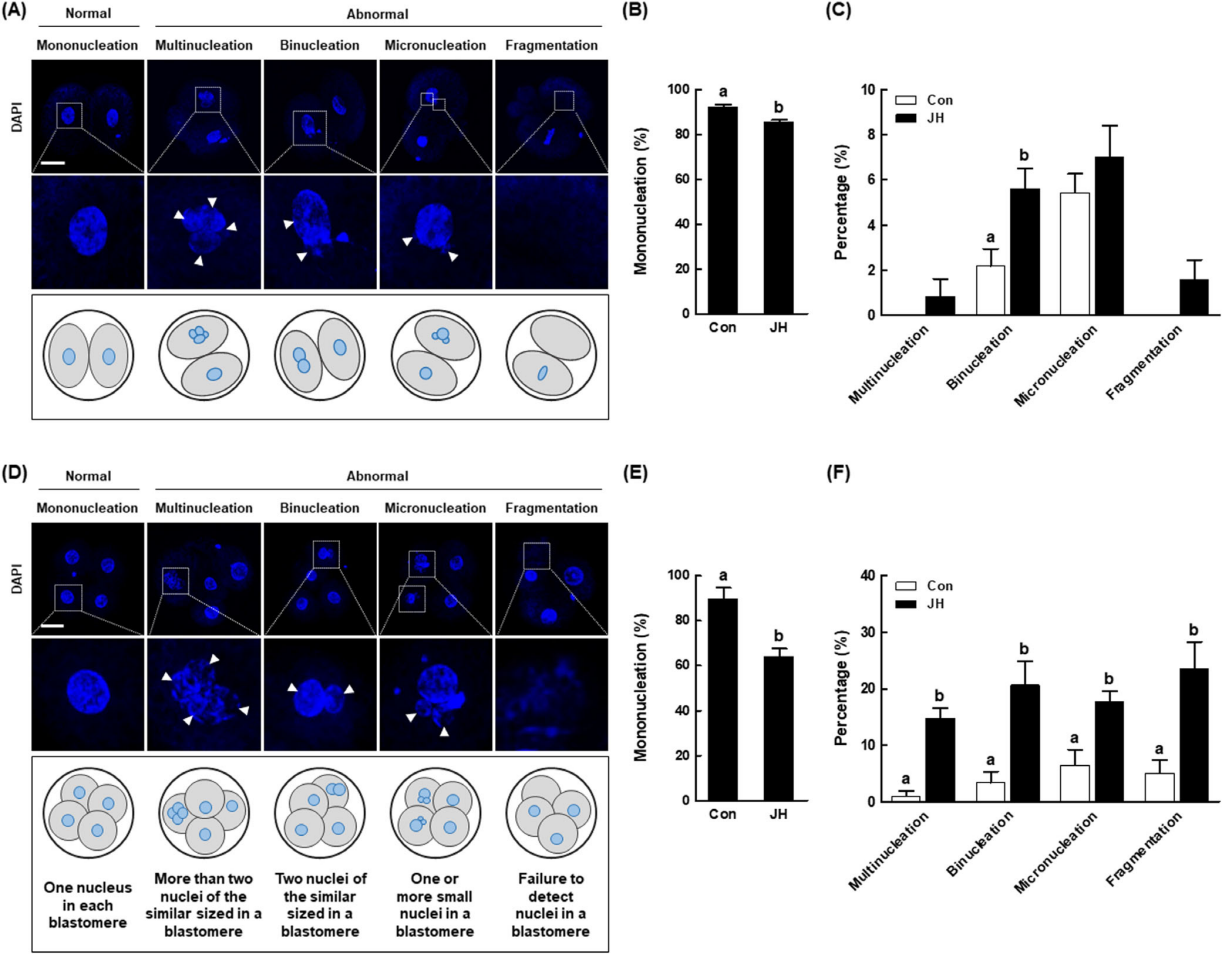
Evaluation of mitotic nuclear abnormalities in NEK2‐suppressed porcine PA embryos. (A) Representative images of normal or abnormal nuclear status in porcine two‐cell stage embryos. Bar = 50 μm. (B) Percentages of mononucleation in the indicated groups (Con; *n* = 123, JH; *n* = 126). (C) Percentages of abnormal nucleation in the indicated groups (Con; *n* = 123, JH; *n* = 126). (D) Representative images of normal or abnormal nuclear status in porcine four‐cell stage embryos. For details, see text. Bar = 50 μm. (E) Percentages of mononucleation in the indicated groups (Con; *n* = 58, JH; *n* = 61). (F) Percentages of abnormal nucleation in the indicated groups (Con; *n* = 230, JH; *n* = 221). Data are derived from at least three independent experiments; different superscripts indicate significant differences (*P* < 0.05).

Errors in mitotic division during embryonic development contribute to the formation of embryos with abnormal nuclear status. Based on the above results, we investigated the effects of NEK2 inhibition on nuclear status in two‐ and four‐cell stage porcine embryos. Among two‐cell stage embryos, the JH group had a significantly smaller proportion of embryos with mononucleation and a larger proportion of embryos with binucleation among embryos exhibiting abnormal nuclear status (Figure 6A–C). Among four‐cell stage embryos, the JH group had a significantly smaller proportion of embryos with mononucleation and a larger proportion of embryos with abnormal nuclear status, such as multinucleation, binucleation, micronucleation and fragmentation (Figure 6D–F). These results suggest that NEK2 inhibition creates porcine embryos with abnormal nuclear status through incomplete chromosome disjunction.

### 3.6 NEK2 inhibition induces DNA damage and early apoptosis in porcine embryos

**FIGURE 7:**
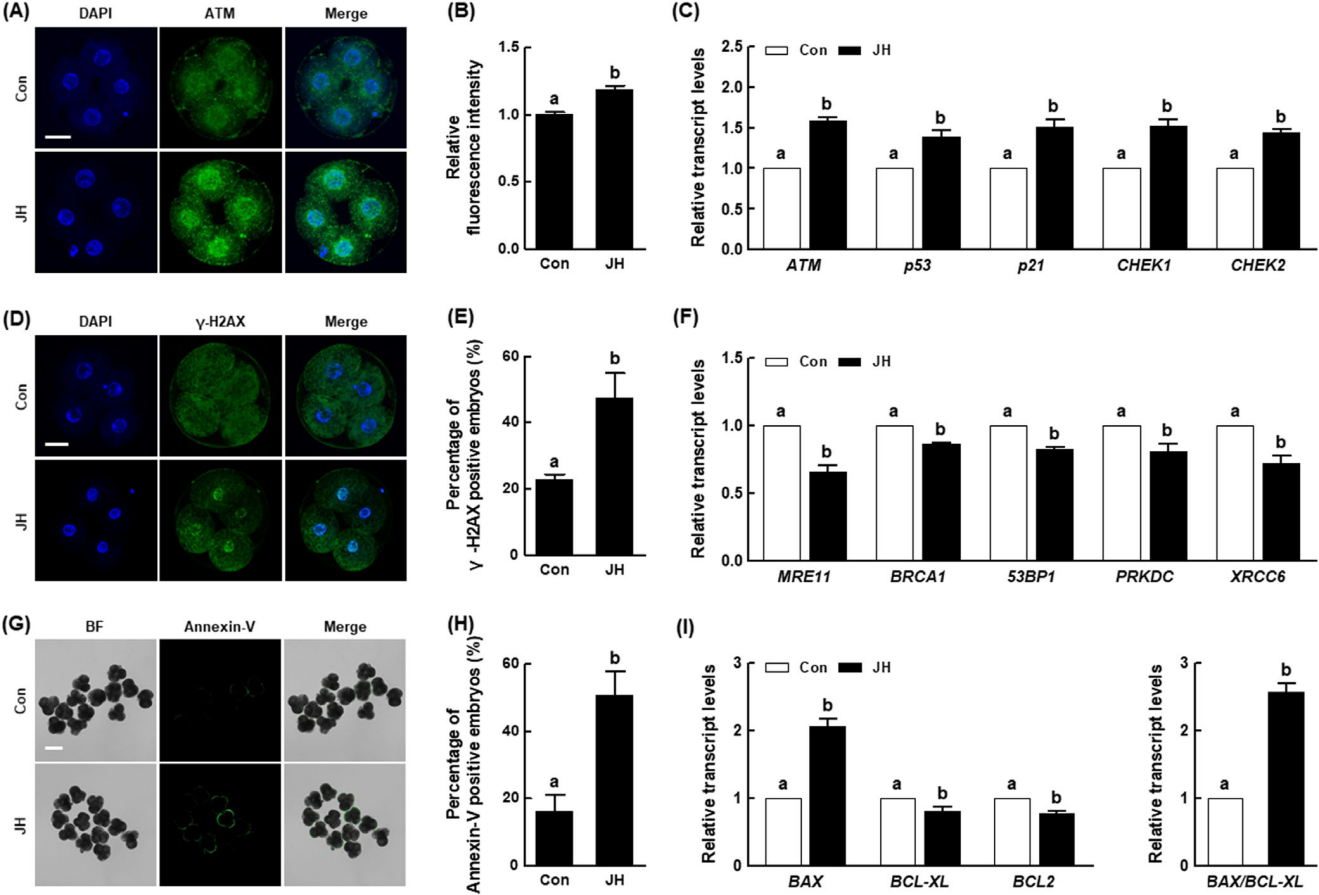
Effects of NEK2 inhibition on DNA damage and early apoptosis in porcine PA embryos (A) Fluorescence images of four‐cell stage embryos stained for ataxia telangiectasia mutated (ATM) (Bar = 50 μm) and (B) quantification of ATM relative fluorescence intensity in the indicated groups (*n* = 36 per group). (C) qRT‐PCR results for transcript levels of DNA damage‐related genes in four‐cell stage embryos (*n* = 3 per group). (D) Fluorescence images of 4‐cell embryos stained for H2AX phosphorylated at Ser 139 (γ‐H2AX) (Bar = 50 μm) and (E) percentages of γ‐H2AX‐positive embryos in the indicated groups (Con; *n* = 48, JH; *n* = 50). (F) qRT‐PCR results for transcript levels of DNA damage repair‐related genes in four‐cell stage embryos (*n* = 3 per group). (G) Fluorescence images of four‐cell stage embryos stained for annexin‐V (Bar = 100 μm) and (H) percentages of annexin‐V‐positive embryos in the indicated groups (Con; *n* = 51, JH; *n* = 53). (I) qRT‐PCR results for transcript levels of apoptosis‐related genes (left) and BAX/BCL‐XL ratio (right) in blastocysts (*n* = 4 per group). Data are derived from at least three independent experiments; different superscripts indicate significant differences (*P* < 0.05).

To determine why NEK2 inhibition delays porcine early embryonic development, we investigated DNA damage and early apoptosis in porcine embryos. The JH group showed significantly higher ATM levels compared with the control; the expression levels of DNA damage‐related genes (*ATM*, *p53* and *p21*) and checkpoint kinase genes (*CHEK1* and *CHEK2*) were upregulated compared with the control (Figure 7A–C). Additionally, compared with the control, the JH group exhibited a significantly larger proportion of γ‐H2AX‐positive embryos; the expression levels of homologous recombination (HR) genes (*MRE11* and *BRCA1*) and non‐homologous end joining (NHEJ) genes (*53BP1*, *PRKDC* and *XRCC6*) were downregulated (Figure 7D–F). Considering that apoptosis is a major consequence of failed DNA damage repair, we investigated whether apoptosis occurred in embryos with NEK2 inhibition. The JH group showed a significantly larger proportion of annexin‐V‐positive embryos compared with the control (Figure 7G,H). Moreover, the expression level of the pro‐apoptosis‐related gene (*BAX*) and the *BAX*/*BCL‐XL* ratio were both significantly upregulated in the JH group, while the anti‐apoptosis‐related genes (*BCL‐XL* and *BCL2*) were significantly downregulated, compared with the control (Figure 7I). These results suggest that NEK2 inhibition causes developmental defects in porcine embryos through disruption of the DNA damage repair system.

### 3.7 NEK2 inhibition disturbs the Wnt/β‐catenin signalling pathway in porcine embryos

**FIGURE 8:**
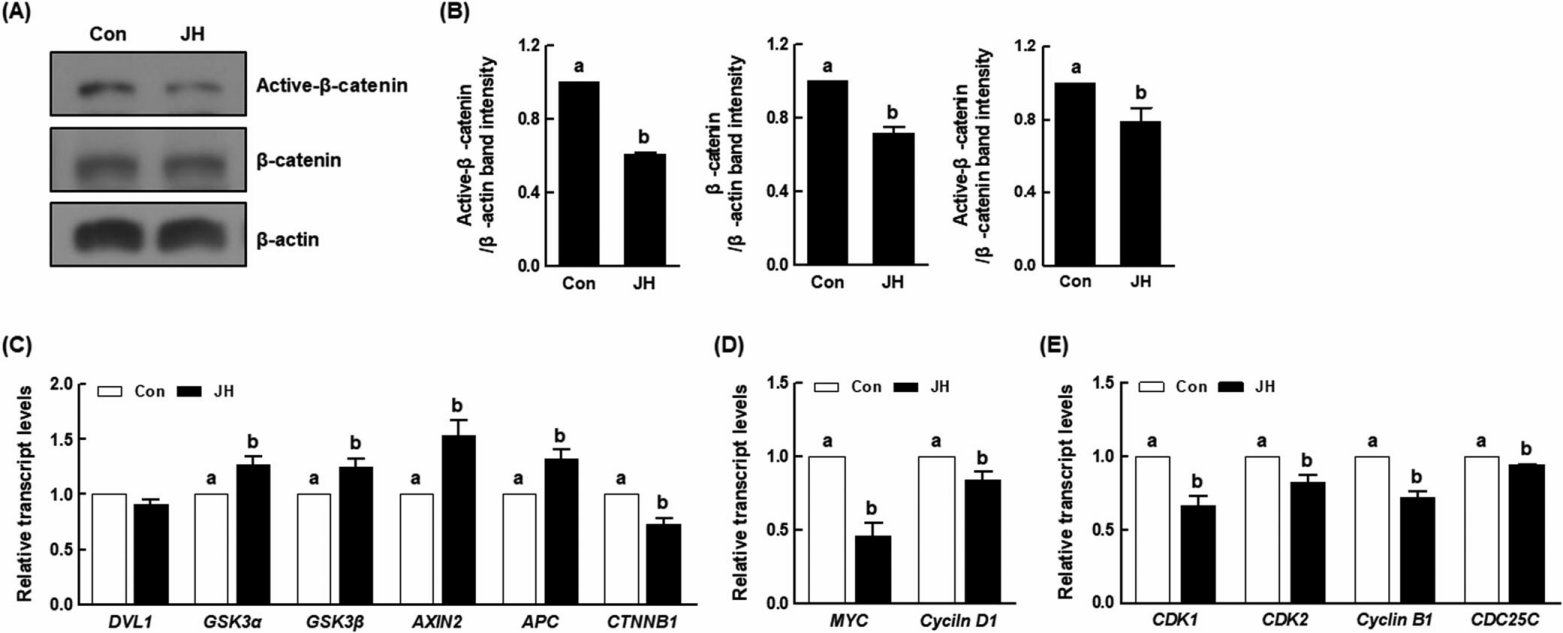
Effects of NEK2 inhibition on Wnt/β‐catenin signalling pathway and cell cycle in porcine PA embryos. (A) Representative images of active‐β‐catenin and β‐catenin Western blotting results and (B) quantification of active‐β‐catenin and β‐catenin band intensity in the indicated groups (*n* = 3 per group). (C) qRT‐PCR results for transcript levels of Wnt/β‐catenin signalling pathway‐related genes (*n* = 4 per group), (D) downstream target genes (*n* = 3 per group) and (E) cell cycle‐related genes in the indicated groups (*n* = 3 per group). Data are derived from at least three independent experiments; different superscripts indicate significant differences (*P* < 0.05).

The Wnt/β‐catenin signalling pathway is a key regulator that participates in various biological processes, including early embryogenesis. To identify the mechanism by which NEK2 inhibition causes developmental defects in porcine embryos, we investigated the expression levels of proteins and mRNAs associated with the Wnt/β‐catenin signalling pathway. NEK2 inhibition resulted in a significant reduction in the protein levels of active‐β‐catenin and β‐catenin compared with the control (Figure 8A,B). Moreover, NEK2 inhibition significantly increased the expression levels of β‐catenin destruction complex‐related genes (*GSK3α*, *GSK3β*, *AXIN2* and *APC*), while reducing the expression levels of the *CTNNB1* gene, compared with the control (Figure 8C). Notably, NEK2 inhibition significantly reduced the expression levels of the Wnt/β‐catenin signalling pathway downstream target genes (*MYC* and *cyclin D1*) and cell cycle‐related genes (*CDK1*, *CDK2*, *cyclin B1* and *CDC25C*) compared with the control (Figure 8D,E), indicating that NEK2 activates this pathway during porcine early embryogenesis.

### 3.8 Activation of the Wnt/β‐catenin signalling pathway rescues NEK2 inhibition‐induced developmental defects in porcine embryos

**FIGURE 9:**
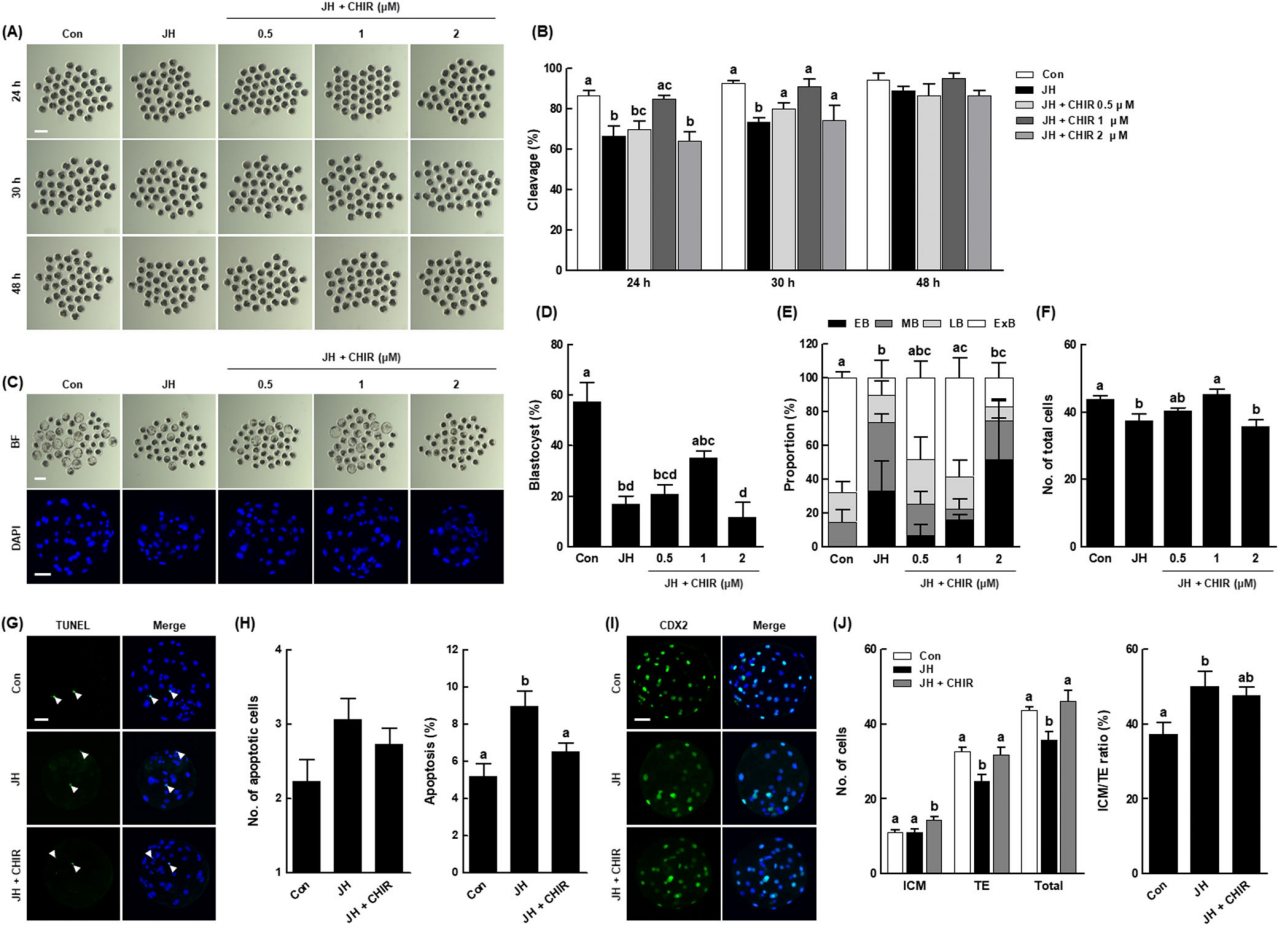
Role of the Wnt/β‐catenin signalling pathway in the effects of NEK2 inhibition on early development and blastocyst quality in porcine PA embryos. (A) Representative images of cleavage and (B) cleavage rates of porcine PA embryos at 24, 30 and 48 h in the indicated groups (*n* = 120 per group). (C) Representative bright‐field images of embryos (upper, Bar = 200 μm) and nuclear‐stained images (lower, Bar = 50 μm) of blastocysts in the indicated groups. (D) Blastocyst formation rates in the indicated groups (*n* = 120 per group). (E) Proportions of various blastocyst stages in the indicated groups (Con; *n* = 66, JH; *n* = 24, 0.5; *n* = 32, 1; *n* = 40, 2; *n* = 24). (F) Total cell numbers in the indicated groups (*n* = 35 per group). (G) Representative images of TUNEL staining of blastocysts (Bar = 50 μm). Embryos were subjected to TUNEL staining (green, white arrow) and nuclear staining (blue). (H) Quantification of the number (left) and proportion (right) of apoptotic cells in the indicated groups (*n* = 30 per group). (I) Representative images of CDX2 staining of blastocysts (Bar = 50 μm). (J) Numbers of ICM, TE and total cells (left), as well as ICM/TE ratios (right), in the indicated groups (*n* = 35 per group). Data are derived from at least three independent experiments; different superscripts indicate significant differences (*P* < 0.05).

To elucidate the interplay between the Wnt/β‐catenin signalling pathway and NEK2 in porcine early embryogenesis, we cultured PA embryos in JH‐treated IVC medium with various concentrations of CHIR (a Wnt activator; 0, 0.5, 1 and 2 μM), then evaluated the rates of cleavage and blastocyst formation. After 24 and 30 h of culture, 1 μM CHIR supplementation restored the JH‐induced cleavage rate to the control level (Figure 9A,B and Table S10). Moreover, the blastocyst formation rate, proportion of expanded blastocysts and total cell number were also restored to control levels via co‐treatment with 1 μM CHIR (Figure 9C–F and Tables S10 and S11). Based on these results, we selected 1 μM CHIR as the optimal concentration and used it for evaluations of blastocyst quality. Although there was no significant difference in the number of apoptotic cells, the increased apoptosis rate was restored to the control level via CHIR co‐treatment (Figure 9G,H and Table S12). Moreover, CHIR co‐treatment significantly increased the numbers of ICM and TE cells compared with the JH group, while modifying the ICM/TE ratio to the control level (Figure 9I,J and Table S13). These results suggest that NEK2 plays an important role in porcine early embryogenesis by activating the Wnt/β‐catenin signalling pathway.

## 4 DISCUSSION

**FIGURE 10:**
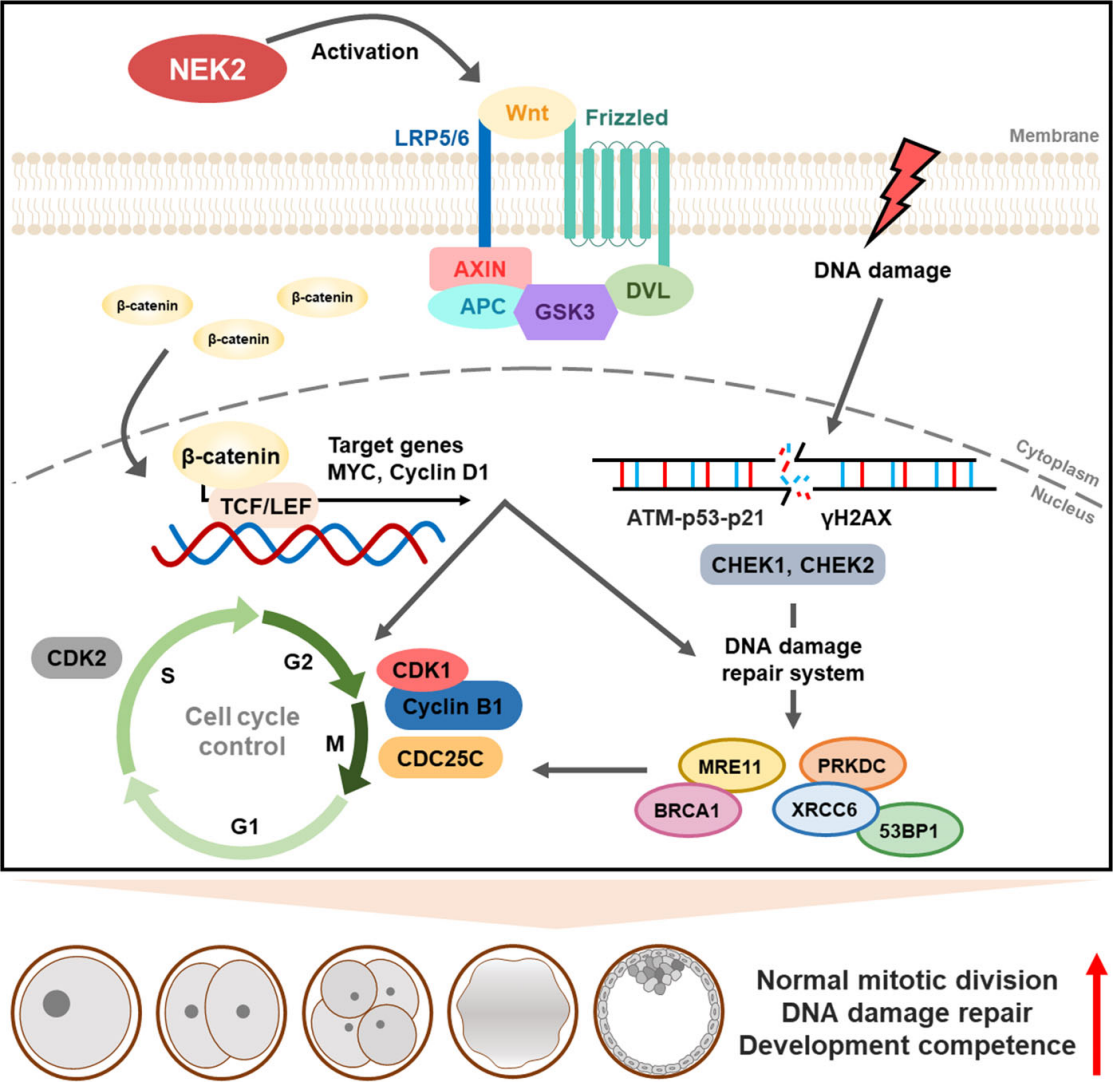
Schematic diagram for NEK2 functions during porcine embryonic development. NEK2 plays an essential roles in normal mitotic division, DNA damage repair and cell cycle progression via the Wnt/β‐catenin signalling pathway in porcine early embryogenesis. Schematic diagram for NEK2 functions during porcine embryonic development. NEK2 plays essential roles in normal mitotic division, DNA damage repair and cell cycle progression via the Wnt/β‐catenin signalling pathway in porcine early embryogenesis.

During early embryonic development, a fertilized embryo undergoes a series of mitotic cell divisions to develop into blastocysts that are capable of implantation in the uterus. In mammals such as humans, 50%–70% of embryos have high rates of developmental arrest due to chromosomal abnormalities; this arrest is likely to result in infertility.^32^ Chromosomal abnormalities mainly arise from DNA damage caused by abnormal cleavage errors. Thus, the preservation of genome integrity is essential for successful preimplantation embryo development. In this study, we investigated the role of NEK2 in porcine embryonic development and identified potential underlying mechanisms. Our findings suggest that NEK2 plays an important role in porcine early embryogenesis by regulating normal mitotic division, DNA damage repair and cell cycle progression via the Wnt/β‐catenin signalling pathway (Figure 10).

In *Aspergillus*, NIMA is responsible for regulating mitotic progression.^33^ NIMA mutations induce G2 arrest, whereas NIMA overexpression causes cells to prematurely enter mitosis; thus, NIMA has a central role in the regulation of G2/M progression.^34^ Moreover, NIMA is important for mitotic events such as chromatin condensation, spindle organization and nuclear envelope formation.^35^ NEKs are involved in regulating several aspects of mitosis because they share an N‐terminal catalytic domain related to NIMA; their C‐terminal non‐catalytic domains are highly divergent, allowing each member to have a distinct function.^36^ NEK2 shares the greatest sequence similarity with NIMA: 47% sequence identity in the catalytic domains.^37^ Previous studies have shown that NEK2 is distributed in the nucleus and cytoplasm of various human cell lines; its expression level exhibits dynamic changes during the cell cycle, reaching peaks in S and G2 phases.^37^, ^38^, ^39^ Moreover, NEK2 mRNA and protein were detected throughout early embryogenesis in mice.^18^ In the present study, we identified the subcellular localization and expression pattern of NEK2 during porcine embryonic development. Our results showed that NEK2 was consistently localized to the nucleus and cytoplasm and expressed in all stages of embryonic development. Additionally, considering that NEK2 serves as a regulator of the cell cycle and mitosis, previous studies have identified a characteristic role for NEK2 in mouse early embryogenesis. There is evidence that NEK2 downregulation via dsRNA microinjection disrupts mouse embryonic development, with arrest confirmed at the four‐cell stage.^18^, ^40^ NEK2 inhibition significantly inhibited the developmental parameters of porcine embryos. Notably, we found that NEK2 inhibition considerably delayed the developmental kinetics of porcine embryos during the preimplantation period, leading to embryonic arrest or death. These results suggest that NEK2 plays an essential role in mammalian early embryogenesis.

Accurate chromosome segregation during mitosis is critical for the maintenance of genomic stability. Errors in chromosome segregation cause chromosomal damage and produce aneuploid or polyploid cells, resulting in embryonic lethality.^41^ NEK2 predominantly localizes to centrosomes, where it orchestrates centrosome separation and bipolar spindle formation. These two events are essential for chromosome segregation into two daughter cells. The segregation process is precisely regulated by the NEK2 kinase function to achieve large‐scale reorganization of cellular components.^13^ Previous studies have revealed that many blastomeres in NEK2‐suppressed mouse embryos showed arrest at interphase with defective nuclear morphology, including dumbbell‐shaped nuclei, nuclear bridges and micronuclei.^18^, ^40^ Consistent with the previous results, we found that NEK2 inhibition delayed normal cell cycle progression during mitotic division and caused abnormal nuclear development in the form of multinucleation, binucleation, micronucleation and fragmentation in two‐ and four‐cell stage embryos. These findings support the notion that NEK2 is crucial for proper chromosome segregation during embryonic development.

Checkpoints are highly evolved processes that detect DNA damage and arrest the cell cycle, allowing cells to progress to the next stage when suitable conditions are present. The major checkpoints are G1/S, Intra‐S, G2/M and spindle assembly.^42^ Among these checkpoints, the G2/M checkpoint actuates ATM and ATR signalling pathways in response to DNA damage and activates checkpoint kinases such as CHEK1 and CHEK2. These changes disable cyclin B1/CDK1, which is responsible for M phase entry, resulting in G2 arrest until the DNA damage is repaired.^43^ p21, a target of the p53 transcription factor, is activated during cellular stress or DNA damage; p53 also participates in the initiation of cell death when DNA damage levels cannot be sufficiently reduced.^44^ Previous studies have shown that NEK2 activation in response to DNA damage delays mitotic entry.^45^ Our results indicate that NEK2 inhibition‐induced DNA damage activates the ATM‐p53‐p21 pathway and increases CHEK1 and CHEK2 levels, ultimately leading to G2 arrest. Additionally, the DNA damage repair mechanism consists of HR and NHEJ. HR repairs damage that occurs in the G2/S phase using sister chromatids as a template, whereas NHEJ is active throughout the cell cycle.^46^ γ‐H2AX contributes to the repair of DNA double‐strand breaks that arise from various injuries, including pathological exposure of DNA ends due to telomere dysfunction. γ‐H2AX suppresses genomic instability during DNA replication and protects against cellular damage through the DNA damage repair systems of HR and NHEJ.^47^ Previous studies have shown that radiation‐induced DNA damage increases γ‐H2AX foci‐positive cells among NEK2‐inhibited cervical cancer cells and reduces the focal positivity of HR regulators involved in DNA repair, confirming that NEK2 knockdown accelerates DNA damage.^29^ The present study showed that NEK2 inhibition increased the proportion of γ‐H2AX‐positive embryos and reduced the expression of genes involved in the HR and NHEJ repair pathways. Thus, NEK2 regulates repair systems in response to DNA damage. Additionally, unrepaired DNA damage leads to the apoptotic pathway and increased positivity for the apoptotic marker annexin‐V.^48^ A previous study revealed that NEK2 overexpression suppressed the expression of pro‐apoptotic genes and upregulated the expression of anti‐apoptotic genes, indicating a possible role for NEK2 in anti‐apoptotic mechanisms.^49^ Our results showed that NEK2 inhibition resulted in a significantly larger proportion of annexin‐V‐positive embryos and altered the expression levels of apoptosis‐related genes, suggesting that NEK2 inhibition‐induced DNA damage is not effectively repaired, leading to cell death.

The Wnt signalling pathway plays a pivotal role during early embryogenesis, in which stabilized β‐catenin activates the transcriptional regulation of TCF/LEF, resulting in the expression of target genes such as MYC and cyclin D1.^50^ Moreover, in the Wnt signalling pathway, β‐catenin, MYC and cyclin D1 regulate DNA damage repair and cell cycle progression.^51^ Previous studies have shown that NEK2 activates the Wnt/β‐catenin signalling pathway and phosphorylates GSK, leading to nuclear accumulation of β‐catenin and the activation of downstream factors such as MYC and cyclin D1.^52^ Consistent with these findings, our results showed that β‐catenin protein levels were reduced in NEK2‐suppressed embryos. Moreover, NEK2 inhibition increased the mRNA levels of genes encoding the β‐catenin destruction complex and decreased the mRNA level of the *CTNNB1* gene. In particular, NEK2 inhibition significantly reduced the expression levels of downstream target genes and cell cycle‐related genes, indicating that NEK2 activates the Wnt/β‐catenin signalling pathway during porcine embryonic development. Intriguingly, the Wnt signalling pathway exhibited distinct expression patterns in vivo and in vitro, and it was downregulated in porcine PA embryos; thus, the abnormal development of porcine PA embryos involved downregulation of the Wnt signalling pathway.^53^ We infer that Wnt signalling pathway activation is important for the in vivo implementation of in vitro embryonic development. CHIR is regarded as an activator of the Wnt signalling pathway, which stabilizes β‐catenin by inhibiting GSK3.^54^ CHIR participates in pluripotency regulation within porcine embryonic stem cells and increases the number of OCT4‐positive cells in blastocysts during porcine embryonic development.^55^, ^56^ The present study showed that CHIR combination treatment restored the rates of cleavage and blastocyst formation that had been reduced by NEK2 inhibition; it also improved blastocyst quality by increasing the numbers of ICM and TE cells. These findings, in conjunction with the results of previous studies, support our interpretation that NEK2 plays an important role in porcine early embryogenesis by activating the Wnt/β‐catenin signalling pathway.

## 5 CONCLUSION

To our knowledge, this study is the first to demonstrate the role of NEK2 in porcine embryonic development and identify potential underlying regulatory mechanisms. NEK2 inhibition significantly reduced developmental competence in terms of the cleavage rate, blastocyst formation rate, cellular survival and cell numbers in porcine embryos. NEK2 inhibition interrupted normal cell cycle progression, including the first mitotic division, and caused abnormal nuclear status through incomplete chromosome disjunction. Moreover, NEK2 inhibition hindered effective DNA damage repair, leading to embryonic arrest or death. Notably, we observed downregulation of the Wnt/β‐catenin signalling pathway in NEK2 inhibited‐embryos; the detrimental effects of NEK2 inhibition on developmental competence were restored by CHIR co‐treatment. These results strongly suggest that NEK2 plays an essential role in mammalian early embryogenesis by regulating DNA damage repair and normal mitotic division via the Wnt/β‐catenin signalling pathway. Our findings provide insights into candidate gene‐governing preimplantation embryonic development for basic reproductive biology and clinical applications involving female infertility.

## AUTHOR CONTRIBUTIONS

Se‐Been Jeon and Pil‐Soo Jeong designed the study, performed experiments, analysed data and wrote the manuscript. Min Ju Kim, Hyo‐Gu Kang, Ji Hyeon Yun, Kyung Seob Lim and Bong‐Seok Song performed experiments and analysed data. Sun‐Uk Kim acquired funding and discussed study. Seong‐Keun Cho discussed study and contributed to data interpretation. Bo‐Woong Sim designed and supervised the study. All authors read and approved the final manuscript.

## FUNDING INFORMATION

This research was supported by the Korea Research Institute of Bioscience and Biotechnology (KRIBB) Research Initiative Program (KGM4252331, KGM5382322), Republic of Korea.

## CONFLICT OF INTEREST STATEMENT

The authors declare no conflicts of interest.

## Supporting information


**Data S1** Supporting Information.

## Data Availability

The data that support the findings of this study are available from the corresponding author upon reasonable request.
