# Supplementary material for: NEK2 plays an essential role in porcine embryonic development by maintaining mitotic division and DNA damage response via the Wnt/β‐catenin signalling pathway
Source: Cell Prolif. 2024 Mar 1;57(8):e13626. doi: 10.1111/cpr.13626 (PMC11294417; doi:10.1111/cpr.13626)
Supplement: Supplementary file 1 — Data S1 Supporting Information. [file CPR-57-e13626-s001.docx]

**NEK2 plays an essential role in porcine embryonic development by maintaining mitotic division and DNA damage response via the Wnt/β-catenin signaling pathway**

Se-Been Jeon^1,2†^, Pil-Soo Jeong^1†^, Hyo-Gu Kang^1,3^, Min Ju Kim^1,2^, Ji Hyeon Yun^1,4^, Kyung Seob Lim^1^, Bong-Seok Song^1^, Sun-Uk Kim^1,5^, Seong-Keun Cho^6*^, and Bo-Woong Sim^1*^

**MATERIALS AND METHODS.**

**Ethics statement**

This study was conducted in strict accordance with the recommendations of the Korea Research Institute of Bioscience and Biotechnology (KRIBB) Institutional Animal Care and Use Committee (approval no. KRIBB-AEC-21261).

**Oocyte collection and *in vitro* maturation (IVM)**

Porcine ovaries were obtained from a local slaughterhouse and transported in 0.9% saline supplemented with 75 μg/mL benzyl-penicillin potassium G and 50 μg/mL streptomycin sulfate at 38.5°C. Cumulus oocyte complexes (COCs) were aspirated from 3–8-mm follicles using a 10-mL syringe with an 18-gauge needle. The COCs were washed with 0.9% saline containing 0.1% bovine serum albumin (BSA; A9647, Sigma-Aldrich, St. Louis, MO, USA) then matured in 500 μL of IVM medium under 5% CO_2_ at 38.5°C. IVM medium consisted of tissue culture medium 199 (M4530, Sigma-Aldrich) supplemented with 10% porcine follicular fluid, 0.57 mM cysteine (C7477, Sigma-Aldrich), 10 ng/mL epidermal growth factor (E9644, Sigma-Aldrich), 25 μM β-mercaptoethanol (M3148, Sigma-Aldrich), 10 IU/mL pregnant mare serum gonadotropin (HOR-272, Prospec, Rehovot, Israel), and 10 IU/mL human chorionic gonadotropin (HOR-250, Prospec). After maturation for 22 h, the medium was removed and COCs were matured for an additional 22 h in the same medium without hormones.

**Parthenogenetic activation (PA), *in vitro* fertilization (IVF), and *in vitro* culture (IVC)**

For parthenogenetic activation, cumulus cells were removed by gentle pipetting in 0.1% hyaluronidase (H3506, Sigma-Aldrich). Oocytes with polar bodies were selected. Selected metaphase II oocytes were exposed to 15 μM of ionomycin (I0634, Sigma-Aldrich) in the dark for 5 min in Dulbecco’s phosphate-buffered saline (DPBS; Gibco, Carlsbad, CA, USA) supplemented with 60 μg/mL gentamicin sulfate salt (G1264, Sigma-Aldrich), 75 μg/mL streptomycin sulfate (S1277, Sigma-Aldrich), and 4 mg/mL BSA. Activated oocytes were then transferred to the post-activation medium consisting of IVC medium (porcine zygote medium-3 with 4 mg/mL BSA) supplemented with 5 µg/mL cytochalasin B and 2 mM 6-dimethylaminopurine for 4 h under 5% CO_2_ at 38.5°C. After 4 h, activated oocytes were transferred to IVC medium at 38.5°C. Porcine zygote medium-3 consisted of 108 mM NaCl (S5886, Sigma-Aldrich), 10 mM KCl (P5405, Sigma-Aldrich), 0.35 mM KH2PO4 (P5655, Sigma-Aldrich), 0.4 mM MgSO4·7H2O (M7774, Sigma-Aldrich), 0.01 mg/mL gentamicin, 25.07 mM NaHCO3 (S5761, Sigma-Aldrich), 0.2 mM sodium pyruvate (P4562, Sigma-Aldrich), 2 mM Ca-lactate2·5H2O (C8356, Sigma-Aldrich), 1 mM L-glutamine (G8540, Sigma-Aldrich), 5 mM hypotaurine (H1384, Sigma-Aldrich).

IVF medium consisted of modified Tris-buffered medium containing 113.1 mM NaCl, 3 mM KCl, 7.5 mM CaCl_2_·2H_2_O (C5670, Sigma-Aldrich), 20 mM Tris (Fisher Scientific, Waltham, MA, USA), 11 mM glucose (G6152, Sigma-Aldrich), 5 mM sodium pyruvate, 2.5 mM caffeine sodium benzoate (C4144, Fluka, Buchs, Switzerland), and 1 mg/mL BSA (A6003, Sigma-Aldrich). Metaphase II oocytes were placed in drops of IVF medium. Fresh swine spermatozoa were washed three times with sperm washing medium (DPBS containing 60 μg/mL gentamicin sulfate salt, 75 μg/mL streptomycin sulfate, and 1 mg/mL BSA). The spermatozoa were resuspended in IVF medium at a final concentration of 1.5 × 10^5^/mL. Next, 2 μL of spermatozoa diluted in IVF medium were added, and the oocytes were co-incubated at 38.5°C for 6 h under 5% CO_2_. After co-culture, spermatozoa attached to the oocytes were stripped by gentle pipetting, and embryos were cultured in IVC medium under 5% CO_2_ at 38.5°C. Cleavage rates were evaluated at 24, 30, and 48 h; blastocyst formation was evaluated at 144 h.

**Chemical treatment**

NEK2 was inhibited using the NEK2-specific inhibitor, JH295 (JH; 4322; Tocris Bioscience, Bristol, UK). JH was dissolved in dimethyl sulfoxide (DMSO) and diluted with IVC medium to final concentrations of 0, 1, 2, and 3 μM. CHIR99021 (CHIR; 4423; Tocris Bioscience), a Wnt activator, was also dissolved in DMSO and diluted with IVC medium to final concentrations of 0, 0.5, 1, and 2 μM. The final DMSO concentration was < 0.2% in IVC medium.

**TdT-mediated dUTP nick-end labeling (TUNEL) assays**

Apoptosis was analyzed using the In Situ Cell Death Detection Kit (Roche, Basel, Switzerland). Formalin-fixed blastocysts were washed three times with DPBS containing 0.1% polyvinyl alcohol (P8136, Sigma-Aldrich) (PVA-PBS), then permeabilized with DPBS containing 1% (v/v) Triton X-100 (T9284, Sigma-Aldrich), for 1 h at room temperature (RT). Next, they were washed three times with PVA-PBS and incubated with fluorescein-conjugated dUTP and terminal deoxynucleotidyl transferase for 1 h at 38.5°C. Subsequently, the blastocysts were washed three times with PVA-PBS and mounted on a glass slide with Vectashield containing 4′,6-diamidino-2-phenylindole (DAPI; Vector Laboratories, Burlingame, CA, USA). The numbers of nuclei and apoptotic cells were observed using a fluorescence microscope (DMi8; Leica, Wetzlar, Germany).

**CDX2 staining**

Formalin-fixed blastocysts were washed three times with PVA-PBS, incubated in DPBS containing 1% Triton X-100 for 1 h at RT, and then washed with PVA-PBS. Next, blastocysts were blocked with PVA-PBS containing 1 mg/mL BSA (1% BSA-PVA-PBS) at 4°C overnight. The blastocysts were blocked with 10% normal goat serum (31873; Thermo Fisher Scientific, Madison, WI, USA) for 1 h and incubated overnight at 4°C with the mouse monoclonal CDX2 antibody (Biogenex Laboratories, Inc., San Ramon, CA, USA). Then, the blastocysts were washed three times with 1% BSA-PVA-PBS and incubated with Alexa-Fluor-488-labeled goat anti-mouse IgG secondary antibody (1:200) at RT for 1 h. Finally, blastocysts were washed three times with 1% BSA-PVA-PBS and mounted on a glass slide in the presence of DAPI. CDX2-positive staining was observed using a fluorescence microscope (DMi8; Leica).

**Cell cycle analysis**

Embryos were collected between 16 and 24 h of culture for the first mitotic division assays. Next, embryos were fixed with formalin solution (HT5011, Sigma-Aldrich) and washed with PVA-PBS. After embryos had been washed, they were mounted on a glass slide with DAPI. The embryos were then classified into interphase, prophase, pro-metaphase, meta-anaphase, and cytokinesis stages, based on the appearances of the nucleus, α-tubulin, and actin.

**Annexin-V staining**

Early apoptosis was assayed using the Annexin-V–FITC Apoptosis Detection Kit. After removal of an embryo’s zona pellucida using acidic Tyrode’s solution, it was fixed in a formalin solution for 2 h and washed with PVA-PBS. Embryos were subsequently incubated in 50 μL of binding buffer containing 5 μL of annexin-V–FITC for 30 min at 38.5°C. Annexin-V staining was immediately observed using a laser scanning confocal fluorescence microscope (LSM700; Zeiss, Oberkochen, Germany).

**Immunocytochemistry**

Embryos or blastocysts were fixed in a formalin solution, washed three times with PVA-PBS, and permeabilized with DPBS containing 1% Triton X-100 for 1 h at RT. The samples were washed three times with PVA-PBS and then stored in blocking medium, which consisted of DPBS containing 0.05% v/v Tween-20 and 2 mg/mL BSA for 1 h at RT. Subsequently, the samples were incubated with primary antibodies to NEK2 (1:500; ab227958; Abcam, Cambridge, MA, USA), α-tubulin (1:500; ab64503; Abcam), and H2AX phosphorylated at Ser 139 (γ-H2AX) (1:200; 2577S; Cell Signaling Technology, Beverly, MA, USA) overnight at 4°C. After samples had been washed three times with DPBS containing 0.05% v/v Tween-20, they were placed in blocking medium for 1 h at RT. After samples had been blocked, they were incubated with secondary antibodies (Alexa Fluor 488-labeled goat anti-rabbit or anti-mouse IgG; 1:200) for 1 h at RT. Finally, the samples were washed three times with DPBS containing 0.05% v/v Tween-20 and mounted on slides with DAPI.

For ataxia telangiectasia mutated (ATM) staining, permeabilized samples were stored in 1% BSA-PVA-PBS for 1 h at RT. Subsequently, the samples were incubated with primary antibody to ATM (1:100; 5883S; Cell Signaling Technology) overnight at 4°C. After samples had been washed three times with 1% BSA-PVA-PBS, they were incubated with secondary antibody (Alexa Fluor 488-labeled goat anti-rabbit IgG; 1:200) for 1 h at RT. Finally, the samples were washed three times with 1% BSA/PVA-PBS and mounted on slides with DAPI.

For actin filament staining, permeabilized samples were stored in blocking medium and then stained with 10 mg/mL phalloidin-tetramethylrhodamine B isothiocyanate at RT for 2 h. After samples had been washed with DPBS containing 0.05% v/v Tween-20, they were subjected to DAPI staining. Signal intensities were observed under a fluorescence microscope (DMi8; Leica) and analyzed using ImageJ software (version 1.47; National Institutes of Health, Bethesda, MD, USA).

**Quantitative real-time polymerase chain reaction (PCR)**

Poly (A) mRNAs were extracted from embryos and blastocysts using the DynaBeads mRNA Direct Kit (Invitrogen Dynal AS, Oslo, Norway), in accordance with the manufacturer’s instructions. Samples were lysed with 100 μL of lysis/binding buffer at RT for 10 min, and mRNA was isolated by adding 30 μL of DynaBeads oligo(dT)25. The beads were separated from the binding buffer using a Dynal magnetic bar (Invitrogen). The bound poly (A) and the beads were washed with washing buffers A and B, to which 7 μL of Tris buffer were added. Subsequently, cDNA was obtained by reverse transcription using the Prime Script RT Reagent Kit with gDNA Eraser (Takara Bio Inc., Shiga, Japan). The resulting cDNA was used as a template for PCR amplification with the following PCR conditions: 95°C for 5 min, then 40 cycles of 95°C for 20 s and 60°C for 20 s. PCR assays were performed with the Mx3000P qPCR system (Agilent Technologies, Santa Clara, CA, USA) and SYBR Premix Ex Taq (Takara Bio Inc.). For the comparative analyses, the expression levels of target genes were quantified relative to that of the H2A. Sample delta Ct ^(SΔCT)^ values were calculated from the difference between the Ct values of H2A and the target genes. Relative gene expression levels between samples and controls were determined using equation 2^−(SΔCT−CΔCT)^. The primers are listed in Table S1.

**Western blotting**

Samples were protease-digested using protein extraction solution (20 mM 4-(2-hydroxyethyl)-1-piperazineethanesulfonic acid [HEPES], 150 mM NaCl, 2 mM egtazic acid [EGTA], 2 mM ethylenediaminetetraacetic acid [EDTA], 20 mM glycerol phosphate, 1% Triton X-100, and 10% glycerol) containing a protease inhibitor cocktail, then boiled at 100°C for 10 min. Samples were separated on a 10% sodium dodecyl sulfate-polyacrylamide gel, transferred to nitrocellulose membranes (HAWP04700; Millipore, Billerica, MA, USA), and blocked with 5 mg/mL BSA in Tris-buffered saline with Tween-20 (TBST) buffer (10 mM Tris-HCl, 150 mM NaCl, and 0.2% Tween-20). Membranes were incubated with primary antibodies to NEK2 (1:1000; ab227958; Abcam), active β-catenin (1:1000; 19807; Cell Signaling Technology), β-catenin (1:1000; 9562; Cell Signaling Technology), and β-actin (1:1000; 66009-1-lg; Proteintech, Rosemont, IL, USA) overnight at 4°C. Next, membranes were washed three times with TBST, blocked with 5% skim milk, and then incubated with horseradish peroxidase-conjugated secondary antibodies (goat anti-rabbit or goat anti-mouse IgG; 1:5000) overnight at 4°C. Specific bands were detected using Super Signal West Femto Maximum Sensitivity Substrate (Thermo Fisher Scientific) and quantified using ImageJ software.

**Time-lapse embryo monitoring**

Embryo cleavage kinetics were assessed using a microwell-culture system and a microscope (DMi8; Leica) equipped with an incubator maintained at 38.5°C under 5% CO_2_. Embryos were placed into each microwell and covered with 20 μL of IVC medium. Images were acquired at 10 min intervals for 48 h. Recorded morphological events included the first and second cleavages.

**Statistical analyses**

All experiments were replicated at least in triplicate. Data are presented as means ± standard errors of the mean. Differences with *P* < 0.05 indicated statistical significance. Differences between two groups were compared using Student’s t-test; differences among three or more groups were compared by one-way analysis of variance, followed by the Tukey–Kramer test. All statistical analyses were performed using SigmaStat software (Systat Software, San Jose, CA, USA).

**Supplementary figure**


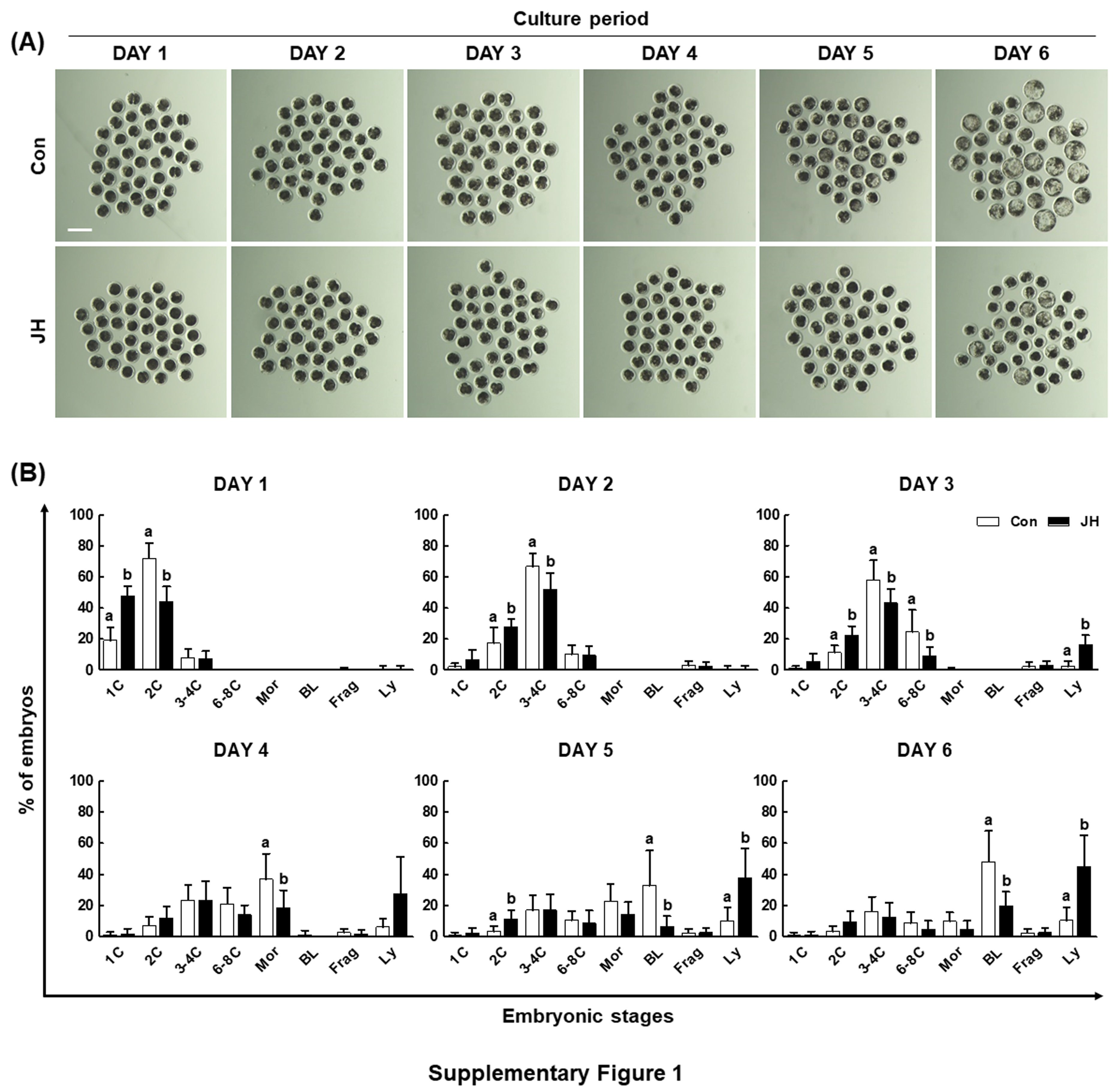


**FIGURE S1.** Effects of NEK2 inhibition on developmental kinetics of porcine PA embryos. (A) Representative images of porcine PA embryos from days 1 to 6 (Bar = 200 µm). (B) Proportions of various embryonic stages on days 1 to 6 in the indicated groups (Con; *n* = 240, JH; *n* = 241). Data are derived from at least three independent experiments; different superscripts indicate significant differences (*P* < 0.05). 1C, one-cell stage; 2C, two-cell stage; 3-4C, three to four cell stage; 6-8C, six to eight cell stage; Mor, morula stage; BL, blastocyst stage; Frag, fragmentation; Ly, lysis


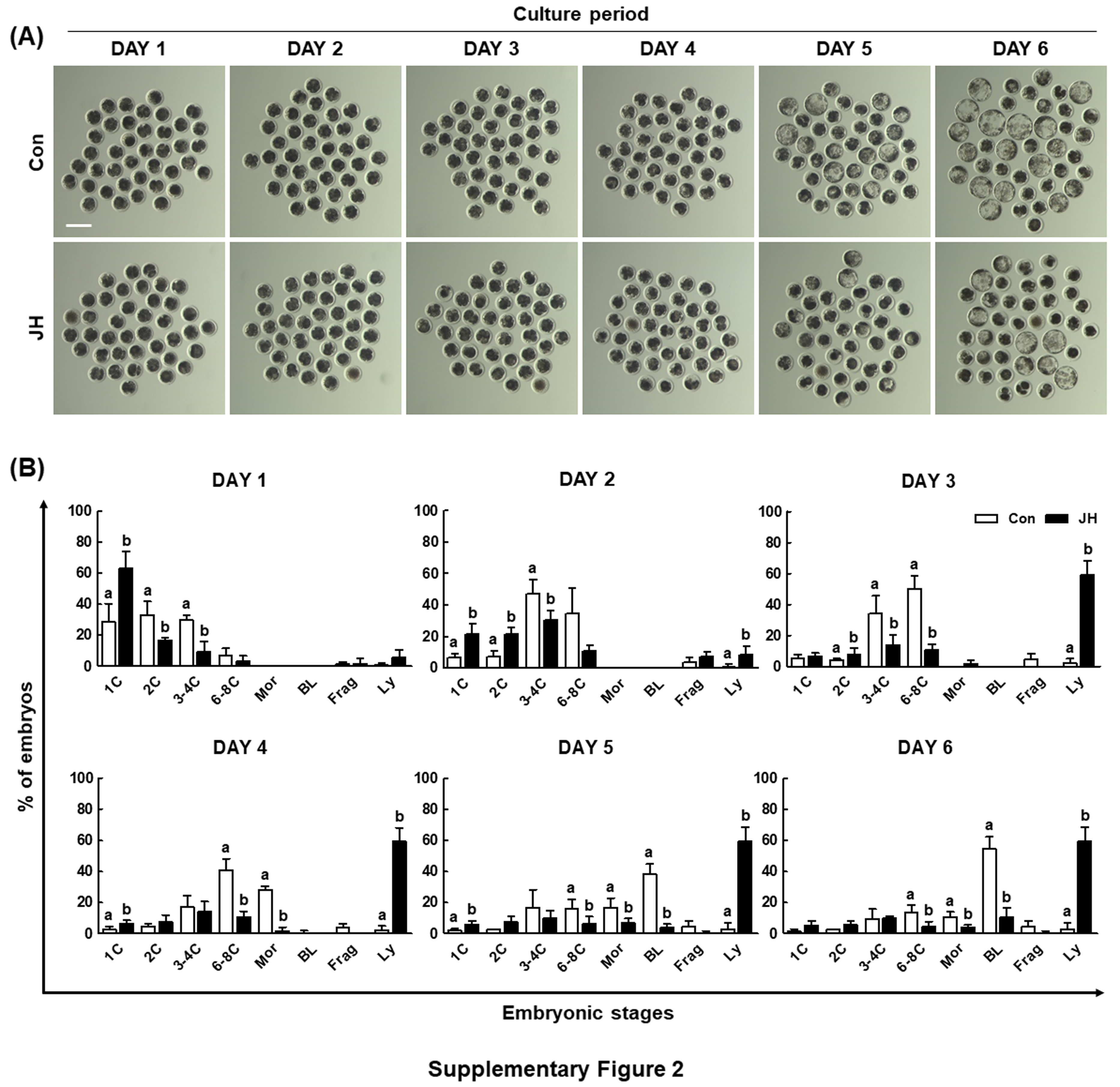


**FIGURE S2.** Effects of NEK2 inhibition on developmental kinetics of porcine IVF embryos. (A) Representative images of porcine IVF embryos from days 1 to 6 (Bar = 200 µm). (B) Proportions of various embryonic stages on days 1 to 6 in the indicated groups (Con; *n* = 152, JH; *n* = 169). Data are derived from at least three independent experiments; different superscripts indicate significant differences (*P* < 0.05). 1C, one-cell stage; 2C, two-cell stage; 3-4C, three to four cell stage; 6-8C, six to eight cell stage; Mor, morula stage; BL, blastocyst stage; Frag, fragmentation; Ly, lysis


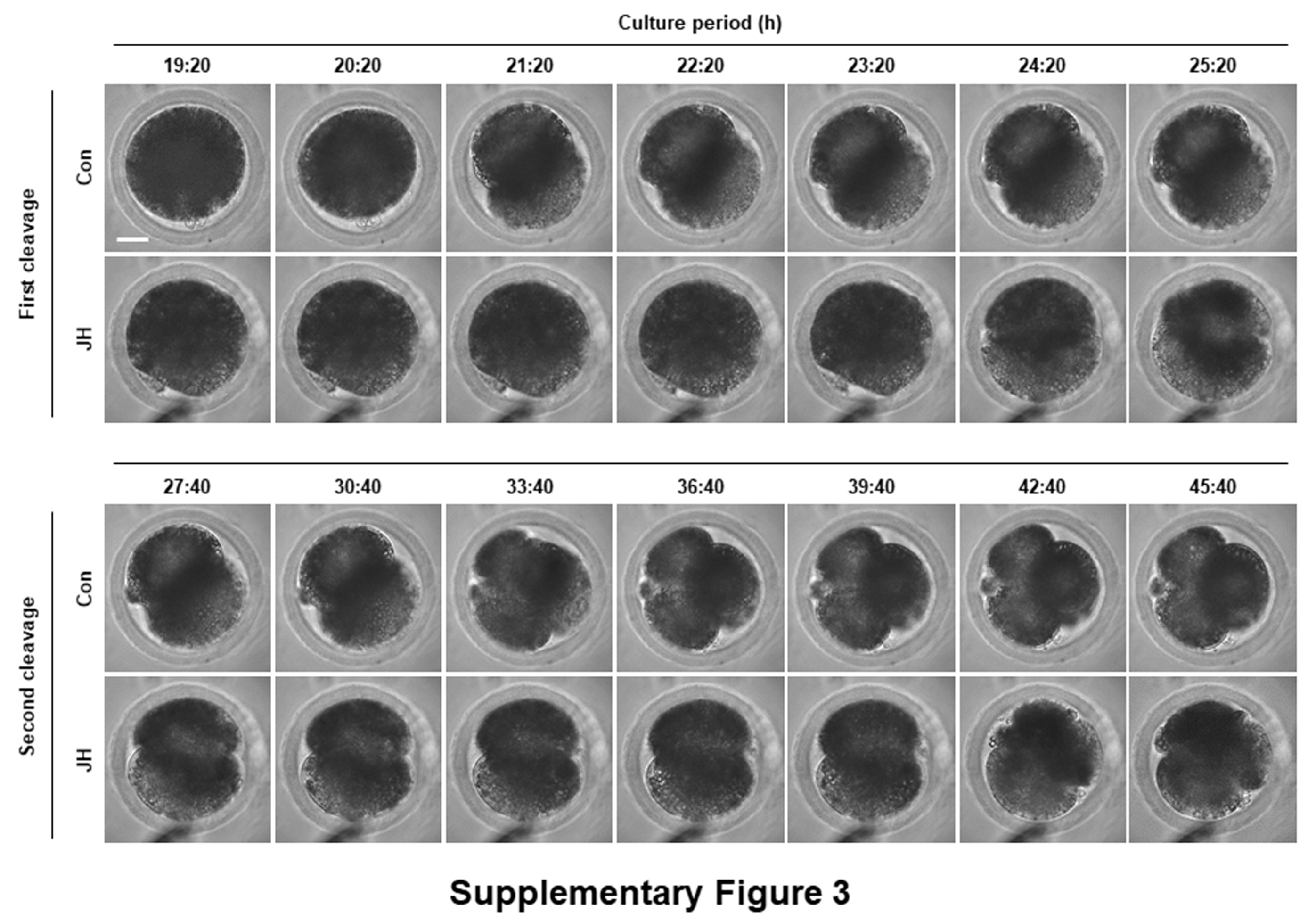


**FIGURE S3.** Effects of NEK2 inhibition on time-lapse monitoring of porcine PA embryos. Representative images of time-lapse monitoring during the first and second cleavages of porcine PA embryos in the indicated groups. Bar = 25 µm.

**Supplementary table**

Table S1. Primer sequences for qRT-PCR

| Gene | Primer sequences | GenBank  accession no. | Product  size (bp) |
| --- | --- | --- | --- |
| *NEK2* | F: 5’- CACTTCAGCGGGGAGAGTAA -3’ | XM_021063863.1 | 100 |
|  | R: 5’- GGAGCCTCCTCTTCAGGTCT -3’ |  |  |
| *OCT4* | F: 5’- AGTGAGAGGCAACCTGGAGA -3’ | NM_001113060.1 | 151 |
|  | R: 5’- ACTGCTTGATCGTTTGCCCT -3’ |  |  |
| *CDX2* | F: 5’- GGCAGCCAAGTGAAAACCAG -3’ | NM_001278769.1 | 119 |
|  | R: 5’- GCCTTTCTCCGAATGGTGAT -3’ |  |  |
| *TEAD4* | F: 5’- GATGTACGGTCGGAATGAG -3’ | NM_001142666 | 145 |
|  | R: 5’- TGCCTGATCCTTTAGCTTGG -3’ |  |  |
| *BAX* | F: 5’- CGATCTCGAAGGAAGTCCAG -3’ | XM_003127290.5 | 251 |
|  | R: 5’- AAGCGCATTGGAGATGAACT -3’ |  |  |
| *BCL-XL* | F: 5’- AGGGCATTCAGTGACCTGAC -3’ | NM_214285.1 | 242 |
|  | R: 5’- TGGATCCAAGGCTCTAGGTG -3’ |  |  |
| *BCL2* | F: 5’- GGAGGGGACACTCTTCTTCC -3’ | XM_021099593.1 | 189 |
|  | R: 5’- CTGGGCACAATTGGTAGCTT -3’ |  |  |
| *ATM* | F: 5’- GTGGAAGAGTTGTGCAAGGG -3’ | NM_001123080.1 | 100 |
|  | R: 5’- GCTCCCCAATGTTCTCAAGC -3’ |  |  |
| *p53* | F: 5’- CCTCACCATCATCACACTGG -3’ | NM_213824.3 | 214 |
|  | R: 5’- GGCTTTCTTCTTTTGCACTGG -3’ |  |  |
| *p21* | F: 5’- CCCCTCTGTCCAGTGAAAGT -3’ | XM_013977858.2 | 103 |
|  | R: 5’- CCAGAAAAGTGCAGGGGAAG -3’ |  |  |
| *CHEK1* | F: 5’- TTCCGGCTTTCTAAGGGTGA -3’ | XM_005667481.3 | 120 |
|  | R: 5’- GCAGATCTATCATGTGGCCG -3’ |  |  |
| *CHEK2* | F: 5’- CCCAGCCTTTCTACAGTCGA -3’ | NM_001137638.1 | 118 |
|  | R: 5’- TCTTTTCATGGTCACCCGGT -3’ |  |  |
| *MRE11* | F: 5’- CAAGAGGCCATGAACAGAGC -3’ | XM_003129788.5 | 107 |
|  | R: 5’- GCCGTCTGTTCTGCCATATC -3’ |  |  |
| *BRCA1* | F: 5’- ACCCTGAGTCTGATCCCTCT -3’ | XM_021066931.1 | 125 |
|  | R: 5’- AGCTGGACTCTTGGCAGATT -3’ |  |  |
| *53BP1* | F: 5’- AACGGCGAAGTAACATCAGC -3’ | XM_021096915.1 | 100 |
|  | R: 5’- GTGGAAGCACGAGGACTTTC -3’ |  |  |
| *PRKDC* | F: 5’- ATCCTTGGCAGAACTTGGGA -3’ | XM_021089419.1 | 110 |
|  | R: 5’- CGGATCGCTCAAAGTTCAGG -3’ |  |  |
| *XRCC6* | F: 5’- TTCCTGGACTTGATGCACCT -3’ | NM_001190185.1 | 115 |
|  | R: 5’- GCGACTCCTCAAAGTGAACC -3’ |  |  |
| *DVL1* | F: 5’- CCGACTTCAAGAACGTGCTC -3’ | XM_003127500.6 | 120 |
|  | R: 5’- GCAGCTTGGCATTATCGTCA -3’ |  |  |
| *GSK3α* | F: 5’- TTTCAGTCCTGGCGAACTCA -3’ | NM_001315708.1 | 160 |
|  | R: 5’- AGTTGTGGCATCAGTCGACT -3’ |  |  |
| *GSK3β* | F: 5’- TGGCTGAACTGTTGCTAGGA -3’ | NM_001128443.1 | 102 |
|  | R: 5’- TTTGCTCCCTTGTTGGTGTC -3’ |  |  |
| *AXIN2* | F: 5’- GACCCCGTGAATCCATACCA -3’ | XM_021066739.1 | 101 |
|  | R: 5’- GAGTCATCCGTCATTGCGTC -3’ |  |  |
| *APC* | F: 5’- GTGATCAGCAAACGCAGGAA -3’ | NM_001206430.1 | 107 |
|  | R: 5’- TGGAACTTCGCTCACAGACT -3’ |  |  |
| *CTNNB1* | F: 5’- AGCAAGCTGATCATTCTGGC -3’ | NM_214367.1 | 103 |
|  | R: 5’- TTCAGTACTCGGCTTGTGGT -3’ |  |  |
| *MYC* | F: 5’- CTGCCAAGAGGGCTAAGTTG -3’ | NM_001005154.1 | 112 |
|  | R: 5’- GTCCGCCTCTTGTCATTCTC -3’ |  |  |
| *Cyclin D1* | F: 5’- CGGGGAGGGTTAGATGTGAT -3’ | XM_021082686.1 | 103 |
|  | R: 5’- CTTTACAAACGGGTGGTGGG -3’ |  |  |
| *CDK1* | F: 5’- CCAGAAATTCGCTTGGCAGG -3’ | NM_001159304.2 | 157 |
|  | R: 5’- AAGTGTGGCCAGAAGTGGAG -3’ |  |  |
| *CDK2* | F: 5’- GTGAGGCGGCAGTAGAAATG -3’ | NM_001285465.1 | 106 |
|  | R: 5’- GGCCTCAGTGAAGCATTGTC -3’ |  |  |
| *Cyclin B1* | F: 5’- GCTAGTGGTGGCTTCAAGGT -3’ | NM_001170768.1 | 100 |
|  | R: 5’- GCGCCATGACTTCCTCTGTA -3’ |  |  |
| *CDC25C* | F: 5’- CCTATGCATCACCAGGACCA -3’ | NM_001123095.1 | 118 |
|  | R: 5’- GGCTCACATCCTTCACGAGT -3’ |  |  |
| *H2A* | F: 5’- AGTTTCCTGTGGGTCGAGTG -3’ | XM_021083382.1 | 162 |
|  | R: 5’- TGCGAGTCTTCTTGTTGTC -3’ |  |  |

F: forward, R: reverse

Table S2. Effects of JH295 (JH) concentrations on *in vitro* development of porcine parthenogenetic activation (PA) embryos

| JH, μM | No. of  embryos examined | Cleavage (%) | | | Blastocyst (%) | Total cell number |
| --- | --- | --- | --- | --- | --- | --- |
|  |  | 24 h | 30 h | 48 h |  |  |
| 0 | 200 | 164 (81.8±2.8)^a^ | 177 (88.1±3.2)^a^ | 188 (93.7±2.3)^a^ | 131 (64.7±7.8)^a^ | 40.8±0.9^a^ |
| 1 | 200 | 156 (78.0±2.0)^a^ | 175 (87.8±2.1)^a^ | 186 (92.8±1.9)^a^ | 101 (50.3±3.6)^a^ | 37.6±0.6^ab^ |
| 2 | 200 | 116 (58.1±3.2)^b^ | 146 (73.0±3.2)^b^ | 187 (93.6±1.1)^a^ | 54 (26.7±3.7)^b^ | 32.9±1.2^b^ |
| 3 | 199 | 81 (40.5±3.8)^c^ | 116 (58.3±1.7)^c^ | 169 (84.9±1.9)^b^ | 7 (3.2±2.7)^c^ | 33.7±2.0^b^ |

Data are the mean ± SEM, and values with different superscript letter within a column differ significantly (*p* < 0.05).

Table S3. Effects of JH on the post-blastulation development of porcine PA blastocysts

| JH, μM | No. of  blastocysts examined | Proportion of blastocysts developed to the following stages (%) | | | |
| --- | --- | --- | --- | --- | --- |
|  |  | Early | Middle | Large | Expanded |
| 0 | 66 | 4.3±2.9 | 16.9±8.4 | 16.4±10.1 | 62.4±3.9^a^ |
| 1 | 58 | 16.6±5.9 | 15.9±8.4 | 20.4±1.9 | 47.1±5.9^ab^ |
| 2 | 25 | 19.5±11.6 | 22.2±3.2 | 33.6±5.9 | 24.6±13.5^bc^ |
| 3 | 14 | 27.7±27.7 | 27.7±5.5 | 38.9±20.0 | 5.5±5.5^c^ |

Data are the mean ± SEM, and values with different superscript letter within a column differ significantly (*p* < 0.05).

Table S4. Effects of JH on cell survival in porcine PA blastocysts

| Groups | No. of blastocysts examined | No. of TUNEL-positive cells | Apoptosis (%) |
| --- | --- | --- | --- |
| Con | 50 | 1.9±0.1 | 4.3±0.2^a^ |
| JH | 50 | 2.0±0.2 | 6.3±0.6^b^ |

Data are the mean ± SEM, and values with different superscript letter within a column differ significantly (*p* < 0.05).

Table S5. Effects of JH on the inner cell mass (ICM)/trophectoderm (TE) ratio in porcine PA blastocysts

| Groups | No. of blastocysts examined | No. of nuclei | | | ICM/TE (%) |
| --- | --- | --- | --- | --- | --- |
|  |  | ICM | TE | Total |  |
| Con | 40 | 11.0±0.7 | 38.7±2.2^a^ | 49.8±2.4^a^ | 32.7±3.0^a^ |
| JH | 40 | 12.1±0.6 | 28.2±2.0^b^ | 40.3±2.4^b^ | 50.4±3.7^b^ |

Data are the mean ± SEM, and values with different superscript letter within a column differ significantly (*p* < 0.05).

Table S6. Effects of JH on *in vitro* development of porcine *in vitro* Fertilization (IVF) embryos

| Groups | No. of  embryos examined | Cleavage (%) | | | Blastocyst (%) | Total cell number |  |
| --- | --- | --- | --- | --- | --- | --- | --- |
|  |  | 24 h | 30 h | 48 h |  |  |  |
| Con | 141 | 73 (68.1±1.3)^a^ | 86 (78.3±3.3)^a^ | 90 (83.5±1.5)^a^ | 67 (47.2±4.1)^a^ | 42.8±1.4^a^ |  |
| JH | 142 | 59 (55.4±1.8)^b^ | 73 (66.6±2.0)^b^ | 83 (76.1±2.1)^b^ | 29 (19.8±5.8)^b^ | 34.2±1.8^b^ |  |

Data are the mean ± SEM, and values with different superscript letter within a column differ significantly (*p* < 0.05).

Table S7. Effects of JH on the post-blastulation development of porcine IVF blastocysts

| Groups | No. of  blastocysts examined | Proportion of blastocysts developed to the following stages (%) | | | |
| --- | --- | --- | --- | --- | --- |
|  |  | Early | Middle | Large | Expanded |
| Con | 66 | 8.9±4.4^a^ | 21.5±8.5 | 22.9±4.7 | 46.5±5.1^a^ |
| JH | 58 | 34.8±7.5^b^ | 24.2±13.2 | 26.5±5.3 | 14.4±5.3^b^ |

Data are the mean ± SEM, and values with different superscript letter within a column differ significantly (*p* < 0.05).

Table S8. Effects of JH on cell survival in porcine IVF blastocysts

| Groups | No. of blastocysts examined | No. of TUNEL-positive cells | Apoptosis (%) |
| --- | --- | --- | --- |
| Con | 47 | 0.9±0.1^a^ | 2.2±0.2^a^ |
| JH | 47 | 1.5±0.1^b^ | 5.1±0.6^b^ |

Data are the mean ± SEM, and values with different superscript letter within a column differ significantly (*p* < 0.05).

Table S9. Effects of JH on the ICM/TE ratio in porcine IVF blastocysts

| Groups | No. of blastocysts examined | No. of nuclei | | | ICM/TE (%) |
| --- | --- | --- | --- | --- | --- |
|  |  | ICM | TE | Total |  |
| Con | 33 | 8.2±0.5 | 33.8±1.5^a^ | 42.0±1.5^a^ | 26.3±2.3^a^ |
| JH | 33 | 9.6±0.8 | 24.6±1.4^b^ | 34.3±1.5^b^ | 45.3±5.1^b^ |

Data are the mean ± SEM, and values with different superscript letter within a column differ significantly (*p* < 0.05).

Table S10. Co-treatment effects of JH and CHIR99021 (CHIR) on *in vitro* development of porcine PA embryos

| Groups,  CHIR, μM | No. of  embryos examined | Cleavage (%) | | | Blastocyst (%) | Total cell number |  |
| --- | --- | --- | --- | --- | --- | --- | --- |
|  |  | 24 h | 30 h | 48 h |  |  |  |
| Con | 120 | 104 (86.6±2.2)^a^ | 111 (92.5±1.4)^a^ | 113 (94.1±3.3) | 69 (57.5±7.6)^a^ | 43.8±1.1^a^ | |
| JH | 120 | 80 (66.6±4.6)^b^ | 88 (73.3±2.2)^b^ | 107 (89.1±1.6) | 20 (16.6±3.3)^bd^ | 37.4±2.0^b^ | |
| JH + CHIR 0.5 | 120 | 84 (70.0±3.8)^bc^ | 96 (80.0±2.8)^a^ | 104 (86.6±5.4) | 25 (20.8±3.6)^bcd^ | 40.2±0.9^ab^ | |
| JH + CHIR 1 | 120 | 102 (85.0±1.4)^ac^ | 109 (90.8±3.6)^a^ | 114 (95.0±2.5) | 42 (35.0±2.8)^abc^ | 45.2±1.6^a^ | |
| JH + CHIR 2 | 120 | 77 (64.1±4.4)^b^ | 89 (74.1±7.4)^a^ | 104 (86.6±2.2) | 14 (11.6±5.8)^d^ | 35.7±1.9^b^ | |

Data are the mean ± SEM, and values with different superscript letter within a column differ significantly (*p* < 0.05).

Table S11. Co-treatment effects of JH and CHIR on the post-blastulation development of porcine PA blastocysts

| Groups,  CHIR, μM | No. of  blastocysts examined | Proportion of blastocysts developed to the following stages (%) | | | |
| --- | --- | --- | --- | --- | --- |
|  |  | Early | Middle | Large | Expanded |
| Con | 66 | 0.0±0.0 | 14.8±7.4 | 17.5±6.1 | 67.6±3.2^a^ |
| JH | 24 | 33.1±17.5 | 40.6±4.9 | 16.0±8.0 | 10.2±10.2^b^ |
| JH + CHIR 0.5 | 32 | 6.6±6.6 | 18.5±7.4 | 26.2±13.5 | 48.5±9.7^abc^ |
| JH + CHIR 1 | 40 | 16.0±3.1 | 6.2±6.2 | 19.3±9.7 | 58.4±11.6^ac^ |
| JH + CHIR 2 | 24 | 51.5±24.4 | 23.0±11.5 | 8.4±4.3 | 16.9±8.6^bc^ |

Data are the mean ± SEM, and values with different superscript letter within a column differ significantly (*p* < 0.05).

Table S12. Co-treatment effects of JH and CHIR on cell survival in porcine PA blastocysts

| Groups | No. of blastocysts examined | No. of TUNEL-positive cells | Apoptosis (%) |
| --- | --- | --- | --- |
| Con | 30 | 2.2±0.2 | 5.2±0.6^a^ |
| JH | 30 | 3.0±0.2 | 8.9±0.8^b^ |
| JH + CHIR | 30 | 2.7±0.2 | 6.5±0.4^a^ |

Data are the mean ± SEM, and values with different superscript letter within a column differ significantly (*p* < 0.05).

Table S13. Co-treatment effects of JH and CHIR on the ICM/TE ratio in porcine PA blastocysts

| Groups | No. of blastocysts examined | No. of nuclei | | | ICM/TE (%) |
| --- | --- | --- | --- | --- | --- |
|  |  | ICM | TE | Total |  |
| Con | 35 | 11.0±0.7^a^ | 32.6±1.1^a^ | 43.6±0.9^a^ | 37.1±3.2^a^ |
| JH | 35 | 11.1±0.8^a^ | 24.6±1.7^b^ | 35.8±2.0^b^ | 49.9±4.0^b^ |
| JH + CHIR | 35 | 14.4±0.8^b^ | 31.7±2.1^a^ | 46.1±2.7^a^ | 47.6±2.1^ab^ |

Data are the mean ± SEM, and values with different superscript letter within a column differ significantly (*p* < 0.05).
